# Metabolic Crosstalk in Triple-Negative Breast Cancer Lung Metastasis: Differential Effects of Vitamin D and E in a Co-Culture System

**DOI:** 10.3390/cancers18020294

**Published:** 2026-01-18

**Authors:** Balquees Kanwal, Saranya Pounraj, Rumeza Hanif, Zaklina Kovacevic

**Affiliations:** 1 Atta-Ur-Rahman School of Applied Biosciences, National University of Sciences and Technology, Islamabad 44000, Pakistan; 2 Tumour Microenvironment Group, Department of Physiology, School of Biomedical Sciences, University of NSW, Sydney, NSW 2052, Australia

**Keywords:** breast cancer, vitamin D, vitamin E, tumour microenvironment, triple-negative breast cancer (TNBC), lung fibroblasts, co-culture model

## Abstract

**Background:** Triple-negative breast cancer (TNBC) is more likely to metastasise to the lungs than other breast cancer (BrCa) types, yet the molecular interactions within the tumour microenvironment (TME) at secondary sites remain poorly understood. **Methods:** This pilot study aimed to explore the metabolic crosstalk between MDA-MB-231 TNBC cells and MRC-5 lung fibroblasts within a co-culture system to replicate the lung metastatic TME. Co-cultures were also treated with Vitamin D or Vitamin E to evaluate the effects of these nutraceuticals on the metabolic crosstalk between TNBC cells and fibroblasts. **Results:** Our findings demonstrate that co-culture induced the activation of fibroblasts into cancer-associated fibroblasts (CAFs), evidenced by increased α-SMA and FAP expression. Metabolic profiling revealed that TNBC cells in co-culture displayed increased expression of enzymes associated with oxidative phosphorylation (OXPHOS) and glutamine metabolism, while fibroblasts exhibited a metabolic profile consistent with glycolysis and lactate metabolism. Vitamin D inhibited lactate metabolism and HIF-1α expression in fibroblasts while suppressing TCA cycle activity in cancer cells, suggesting a potential role in disrupting oncogenic metabolic crosstalk. Conversely, Vitamin E treatment was associated with increased expression of TCA cycle and oxidative metabolism-related markers in BrCa cells without significantly affecting fibroblast glycolysis. Such differential metabolic responses may contribute to metabolic heterogeneity within the tumour microenvironment. **Conclusions:** These results provide valuable insights into the metabolic dynamics of TNBC metastases in the lung TME and demonstrate that Vitamins D and E exert distinct effects on metabolic crosstalk between cancer cells and fibroblasts. These findings may have significant implications for the potential supplementation of Vitamins D and E in patients with metastatic TNBC and justify further in-depth analysis.

## Simple Summary

Triple-negative breast cancer (TNBC) is more likely to spread to the lungs than other types of breast cancer, yet the interplay between cancer cells and the lung microenvironment is not well characterised. In this study, a co-culture system of TNBC cells and lung fibroblasts was employed to study their metabolic interactions. The effects of Vitamin D and Vitamin E on these interactions were also assessed. Fibroblasts showed increased supportive characteristics toward cancer cells, while cancer cells exhibited alterations in energy and nutrient metabolism. Vitamin D appeared to reduce some of these supportive metabolic activities in fibroblasts and cancer cells, whereas Vitamin E was associated with increased metabolic activity in cancer cells. These observations provide insights into how cancer cells and lung fibroblasts interact and suggest that Vitamins D and E may influence these metabolic interactions, warranting further investigation in metastatic TNBC.

## 1. Introduction

Breast cancer (BrCa) is the most commonly diagnosed cancer in females worldwide, and 90% of BrCa-related mortalities are associated with metastasis [1]. Triple-negative breast cancer (TNBC) accounts for approximately 15% of all invasive BrCa and exhibits the highest metastatic potential [2], with lungs being the major metastatic sites for TNBC [3]. Within the lung tumour microenvironment (TME), cancer cells secrete soluble factors that significantly alter the phenotype of surrounding stromal cells. These factors drive pro-tumourigenic processes, including the transformation of resident lung fibroblasts into cancer-associated fibroblasts (CAFs), which play a pivotal role in driving tumour growth, angiogenesis, and immune suppression [3,4,5,6,7,8,9]. However, the metabolic changes that shape the lung metastatic niche to enable TNBC outgrowth require further elucidation. TNBC displays strong propensity for lung metastasis, where lung associated fibroblast act as a major stromal component that actively contributes to the metastatic microenvironment [10]. The lung microenvironment is metabolically different because of high oxygen availability and active nutrient exchange, which may particularly benefit oxidative and glutamine-mediated metabolism in metastatic TNBC cells [11]. Therefore, the complex metabolic crosstalk between TNBC cells and lung fibroblasts remains poorly characterised, yet is crucial to understand in order to develop more effective therapeutics against metastatic TNBC [12].

Metabolic re-programming is a hallmark of cancer [13], with cancer cells re-wiring their metabolic pathways to enable survival in oxygen and nutrient-depleted environments [14]. To achieve this, cancer cells enhance aerobic glycolysis to produce more pyruvate and lactate, which are then diverted away from the tricarboxylic acid (TCA) cycle and used for production of biomass and nucleotides required for cell division [15,16]. However, as the TCA cycle is a metabolic hub for energy metabolism and redox balance, cancer cells also adapt mechanisms to maintain oxidative phosphorylation (OXPHOS) [17,18]. To achieve this, cancer cells become dependent on glutamine for replenishing intermediates of the TCA cycle [19]. Glutamine, the most abundant amino acid in the human body, is taken up by cancer cells and converted into the TCA intermediate, α-ketoglutarate (α-KG), in a process known as glutaminolysis [20]. Cancer cells increase the expression of enzymes involved in glutaminolysis and up-regulate glutamine transporters to enable uptake of large quantities of glutamine from the TME [21].

However, metabolic re-programming is not limited to cancer cells, with surrounding CAFs also undergoing significant metabolic changes. Cancer cells induce oxidative stress in neighbouring CAFs, leading to increased expression of hypoxia induced factor 1 (HIF-1α), a master metabolic regulator that promotes anaerobic glycolysis [22]. This leads to increased production of metabolic intermediates such as pyruvate and lactate, which are secreted into the TME and taken up by cancer cells to fuel their TCA cycle [15]. As a result, metabolic crosstalk between cancer cells and fibroblasts plays an important role in driving tumour progression and is a promising therapeutic target in BrCa [23].

Recent studies demonstrated that nutraceuticals including Vitamin D (VD) and Vitamin E (VE) can influence BrCa metabolism and may potentially regulate the TME [24,25,26]. Deficiency of VD was linked to increased BrCa risk, with VD supplementation shown to suppress HIF-1 transcriptional activity, reduce the risk of metastasis [27,28], and inhibit CAF activation [24]. VD was also found to inhibit key metabolic pathways in BrCa, including glycolysis and glutaminolysis, by down-regulating crucial enzymes involved in these processes, such as Glucose transporter 1 (GLUT1), Hexokinase II (HKII), lactate dehydrogenase A (LDHA), and sodium-dependent transporter of amino acids (SLC1A5) [29,30]. Vitamin E, specifically α-tocopherol, a fat-soluble antioxidant, plays a crucial role in modulating oxidative stress and BrCa metabolism [31], particularly by down-regulating key enzymes like HKII and succinate dehydrogenase B (SDHB) [26]. Both vitamins therefore hold potential for impacting metabolic activity within the metastatic niche, suggesting a novel area for therapeutic exploration.

In this pilot study, we investigated the metabolic interactions within a model of the metastatic lung microenvironment by co-culturing MDA-MB-231 TNBC cells with MRC-5 lung fibroblasts. Our results reveal significant metabolic re-programming in both cell-types upon co-culture. We also demonstrated that supplementation with either VD or VE influenced the metabolic interactions between these cells, although the effects of these nutraceuticals were markedly different. This study is the first to reveal the effects of VD and VE on metabolic crosstalk in the context of metastatic TNBC and justifies further in-depth investigation into the potential use of VD as a supplement in TNBC patients.

## 2. Materials and Methodology

### 2.1. Materials

MDA-MB-231 and MRC-5 cells were purchased from the American Tissue Culture Collection (ATCC; Rockville, MD, USA). Vitamin D (1α,25-Dihydroxyvitamin D3) and Vitamin E (α-Tocopherol) analogues were sourced from Sigma-Aldrich (St. Louis, MO, USA). The primary antibodies used in this study, including α-SMA (Cat.#:19245S), FAP (Cat.#:66562S), GLUL (Cat.#:80636S), GDH (Cat.#:12793S), ASCT2 (Cat.#:5345S), Aconitase (Cat.#:6571T), SDHA (Cat.#:11998T), CS (Cat.#:14309T), IDH2 (Cat.#:56539T), Fumarase (Cat.#:4567T), MPC2 (Cat.#:46141S), GLUT1 (Cat.#:73015S), HKII (Cat.#:2867S), PKM2 (Cat.#:4053T), PDH (Cat.#:3205S), LDHA (Cat.#:2012S), MCT1 (Cat.#:76508S), HIF-1α (Cat.#:36169S), and c-Myc (Cat.#:5605S) were from Cell Signalling Technologies (CST; Danvers, MA, USA). MCT4 (Cat.#:ab234728) was from Abcam, UK. Secondary antibodies used included anti-mouse (Cat.#:A4416) from Sigma-Aldrich and anti-rabbit (Cat.#:7074P2) from CST. Anti-β-actin antibody (Cat.#:A1978; Sigma-Aldrich) was utilised as a loading control. Antibody dilutions were used as per the manufacturer’s instructions.

### 2.2. Cell Culture Conditions

MDA-MB-231 cells were cultured in Dulbecco’s modified eagle medium (DMEM), while MRC-5 fibroblasts were cultured in minimum essential media (MEM), with both media supplemented with 10% FBS, 1% penicillin/streptomycin, 1% sodium pyruvate, and 1% *v/v* MEM non-essential amino acids (ThermoFisher Scientific, Waltham, MA, USA). Cells were kept at 37 °C in a humidified 5% CO_2_ incubator and media-changed every 3 days. Both cell lines were regularly tested for mycoplasma by MycoAlert mycoplasma detection kit (Lonza) and certified mycoplasma free.

### 2.3. Co-Culture Experiments and Treatments

MDA-MB-231 and MRC-5 cells were co-cultured using Transwell^®^ permeable 24 mm inserts (0.4 μm pore Polyester membrane) placed into 6-well plates. Briefly, each cell line was seeded separately in its respective medium, either in wells or inserts at 65–70% confluency, and incubated for 24 h to allow attachment. Inserts containing one cell type were then placed into wells containing the other cell type, allowing the diffusion of soluble factors, metabolites and cytokines through the porous membrane enabling metabolic and paracrine interactions between cells. Co-cultures were incubated for an additional 72 h. To assess the effects of VD or VE, 20 nM VD [32] or 20 µM VE [31] was added to the co-culture media for the entire 72 h incubation.

### 2.4. Protein Extraction

Cells were lysed using lysis buffer (10 mM Tris buffer, 150 mM NaCl, 0.5% SDS, 1% Triton X-100, 1 mM EDTA, 0.04 mM NaF, 4% protease inhibitor cocktail, and 2% phosphatase inhibitor cocktail), followed by sonication at 20 kH and centrifugation (14,000× *g*; 40 min at 4 °C). Protein concentration of the supernatant was measured using Pierce’s BCA Protein Assay (ThermoFisher) and absorbance measured at 562 nm using a microplate reader (SpectraMax M3, San Jose, CA, USA).

### 2.5. Western Blot Analysis

Western blotting was performed as described previously [33]. Briefly, proteins were separated on 10% SDS-PAGE gels, transferred to PVDF membranes (BioRad, Gladesville, NSW, Australia), followed by incubation with primary antibodies overnight at 4 °C. Following washes with Tris-buffered saline containing 0.1% Tween 20 (TBST), membranes were incubated with secondary antibodies for 1 h at room temperature. Bands were visualised using an ECL kit (Merck, Rahway, NJ, USA) and the ChemiDoc imaging system (BioRad, Australia).

### 2.6. Densitometry and Statistical Analysis

Densitometric analysis of Western blots were performed using Quantity One software (Version 4.6.7) and ChemiDoc ImageJ software (Version 3.0.1; BioRad, Hercules, CA, USA) and normalised to the β-actin loading control. Data are expressed as the mean ± SD from three independent replicates. Statistical significance was assessed using a paired Student’s *t*-test for predefined comparisons between treatment and control groups for each protein, with *p* ≤ 0.05 considered statistically significant.

## 3. Results

### 3.1. MDA-MB-231 Cells Participated in Metabolic Crosstalk with MRC-5 Fibroblasts

To determine how co-culture of MDA-MB-231 cell with MRC-5 fibroblasts influences metabolic crosstalk between these two cell types, cells were cultured separately or co-cultured together in transwell plates for 72 h followed by protein extraction from both cancer cells and fibroblasts. The cancer cells cultured alone were designated “CC”, while cancer cells co-cultured with fibroblasts were labelled “CC^(FB)^”. Conversely, fibroblasts cultured alone were labelled “FB”, while fibroblasts co-cultured with cancer cells were labelled “FB^(CC)^”.

**Figure 1:**
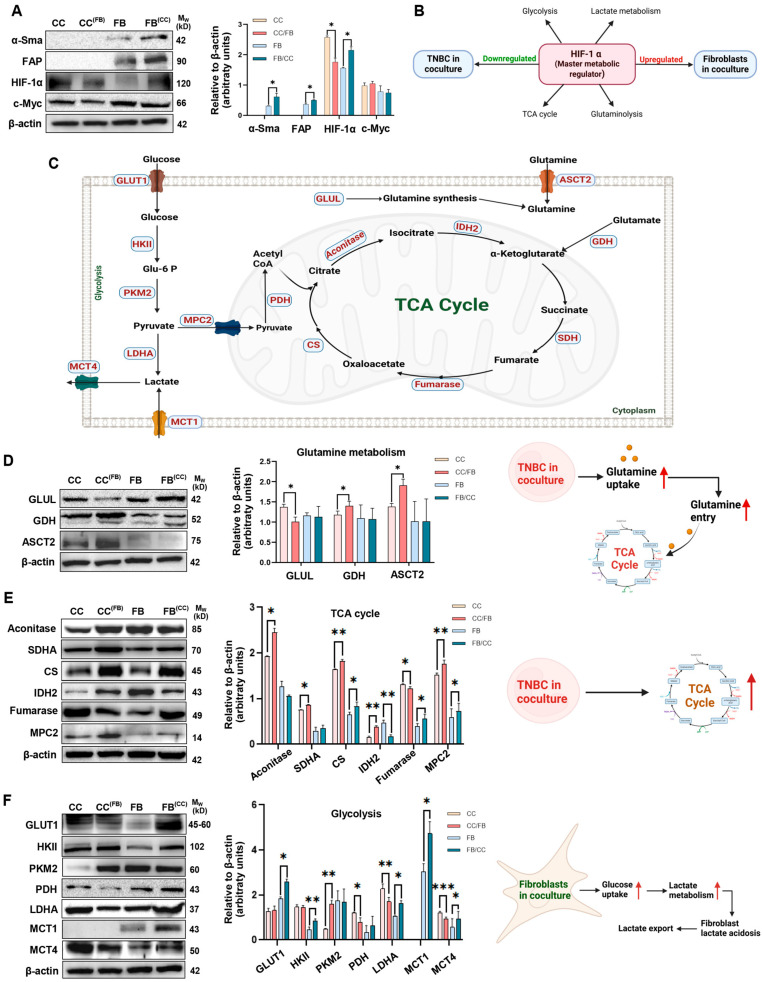
(**A**) MDA-MB-231 and MRC-5 cells were co-cultured for 72 h and both cell lines were assessed via Western blot for CAF activation markers (α-SMA and FAP) and master metabolic regulators (HIF-1 α and c-Myc); (**B**) A schematic representation depicting HIF-1α as the master regulator of metabolism, regulating key metabolic pathways and showing its differential expression in cancer cells and fibroblasts within the co-culture system; (**C**) A general scheme of cellular energy metabolism is shown, highlighting key enzymes (indicated in boxes) whose expression levels were evaluated in the co-culture experiments. Co-cultures were assessed for (**D**) glutamine metabolic enzymes (GLUL, GDH and ASCT2); (**E**) TCA cycle enzymes (Aconitase, SDHA, CS, IDH2, fumarase and MPC2); (**F**) glycolysis and other metabolic enzymes (GLUT1, HKII, PKM2, LDHA, PDH, MCT1, and MCT4). (**A**,**D**–**F**) Results are represented as mean ± SD (*n* = 3). Significance relative to control cells: * *p* < 0.05, ** *p* < 0.01, *** *p* < 0.001. The uncropped original Wester blotting images can be found in File S1.

The effect of co-culture on activation of MRC-5 fibroblasts into CAFs was assessed by examining well-established CAF markers α-SMA and FAP [34,35]. Co-culture significantly increased the expression of both activation markers in MRC-5 fibroblasts, providing marker-based evidence of fibroblast activation towards a CAF-like phenotype (Figure 1A). We next assessed the expression of master regulators of metabolism, namely HIF-1 α and c-Myc in both MDA-MB-231 and MRC-5 cells following co-culture. While HIF-1α expression was reduced in cancer cells, its levels were significantly up-regulated in fibroblasts following co-culture (Figure 1A). In contrast, c-Myc expression remained unchanged in both cancer cells and fibroblasts (Figure 1A).

Hypoxia, a hallmark of tumours, positions HIF-1 α as a master regulator of tumour metabolism by orchestrating key metabolic processes, including glucose, glutamine, nucleotide, and lipid metabolism by directly influencing the expression of numerous metabolic enzymes [36] (Figure 1B)**.** Hence, further studies focused on examining specific enzymes involved in these metabolic pathways to determine their expression in MDA-MB-231 and MRC-5 cells following co-culture. These include enzymes involved in glutamine metabolism (GLUL, GDH, and ACT2), glycolysis (GLUT1, HKII, and PKM2), the TCA cycle (Aconitase, SDHA, CS, IDH2, Fumarase, MPC2), and other metabolic enzymes (LDHA, PDH, MCT1, and MCT4) (Figure 1C).

### 3.2. Glutamine Fuels MDA-MB-231 Cells in Co-Culture to Generate Energy Through the TCA Cycle

Examining glutamine metabolic enzymes, GLUL was significantly reduced in MDA-MB-231 cells when co-cultured with fibroblasts (Figure 1D). The expression of GDH, which enables glutamine entry into the TCA cycle [37], was significantly up-regulated in cancer cells, while remaining unaffected in fibroblasts following co-culture (Figure 1D). Interestingly, the glutamine transporter ASCT2 was significantly elevated in cancer cells upon co-culture with fibroblasts, providing marker-based evidence consistent with enhanced glutamine uptake (Figure 1D). These findings suggests that glutamine metabolism in TNBC cells may be affected by co-culture with fibroblasts, as characterised by marker-based evidence of decreased glutamine synthesis and increased glutamine uptake toward the TCA cycle.

The enzymes driving the TCA cycle including Aconitase, SDHA, and IDH2 were significantly up-regulated in MDA-MB-231 cells co-cultured with fibroblasts (Figure 1E). Further, CS was also up-regulated in both cancer cells and fibroblasts upon co-culture. Despite being increased in cancer cells following co-culture, IDH2 was significantly reduced in fibroblast under these same conditions. Conversely, Fumarase was significantly reduced in MDA-MB-231 cells, while being significantly enhanced in fibroblasts upon co-culture. MPC2, which enables entry of pyruvate into mitochondria, was increased in both MDA-MB-231 and MRC-5 cells when co-cultured (Figure 1E). Overall, these results suggest a remodelling of TCA cycle-associated metabolic enzyme expression in MDA-MB-231 cancer cells following co-culture with fibroblasts. This is consistent with alterations in oxidative and glutamine-associated metabolic pathways, as reflected by changes in enzyme expression patterns.

### 3.3. Exposure to Cancer Cells Potentially Enhanced Glycolysis and Lactate Production by Fibroblasts

In fibroblasts, both GLUT1 and HKII were significantly elevated following co-culture (Figure 1F), suggesting that fibroblasts have enhanced glucose uptake and glycolysis following exposure to MDA-MB-231 cells. PKM2, the enzyme catalysing the last step in glycolysis and promoting the conversion of Glu-6-P to pyruvate, was significantly elevated in cancer cells following co-culture, while its expression remained unchanged in fibroblasts. However, the mitochondrial enzyme PDH, which converts pyruvate into Acetyl-CoA for entry into the TCA cycle, was significantly down-regulated in cancer cells following co-culture (Figure 1F), indicating reduced enzymatic capacity for pyruvate entry into TCA cycle under co-culture conditions and potentially reflecting a shift in pyruvate use.

Apart from entry into the TCA cycle, the other fate of pyruvate is conversion into lactate by the enzyme LDHA, followed by export from cells via the MCT4 transporter [38]. Notably, both LDHA and MCT4 expression were also reduced in cancer cells following co-culture (Figure 1F), reflecting a coordinated down-regulation of the pathways for lactate metabolism. Together, these changes in enzyme expression suggest an altered regulation of pyruvate-utilising pathways in cancer cells under co-culture conditions. Analysis of LDHA and MCT4 in fibroblasts revealed significant up-regulation of both enzymes after co-culture (Figure 1F). Further, the lactate transporter MCT1, which was only detected in fibroblasts, was also up-regulated following co-culture with cancer cells (Figure 1F). These changes, together with the elevated GLUT1 and HK2, are indicative of increased glycolytic activity in co-cultured fibroblasts and may reflect enhanced lactate production and its export into the TME.

### 3.4. VD Down-Regulates HIF-1α, the Master Regulator of Metabolism, in Co-Cultured Fibroblasts

Given the significant metabolic changes in MDA-MB-231 cells and fibroblasts upon co-culture, we next examined whether these metabolic alterations are influenced by VD. To assess how VD influences metabolic crosstalk between MDA-MB-231 cells and MRC-5 fibroblasts, VD was added to the co-culture media for the entire 72 h incubation period and its effects were examined on both cancer cells (CC^(FB)^) and fibroblasts (FB^(CC)^) in co-culture. Notably, cancer cells or fibroblasts incubated alone with no VD treatment served as the relevant controls (Control).

**Figure 2:**
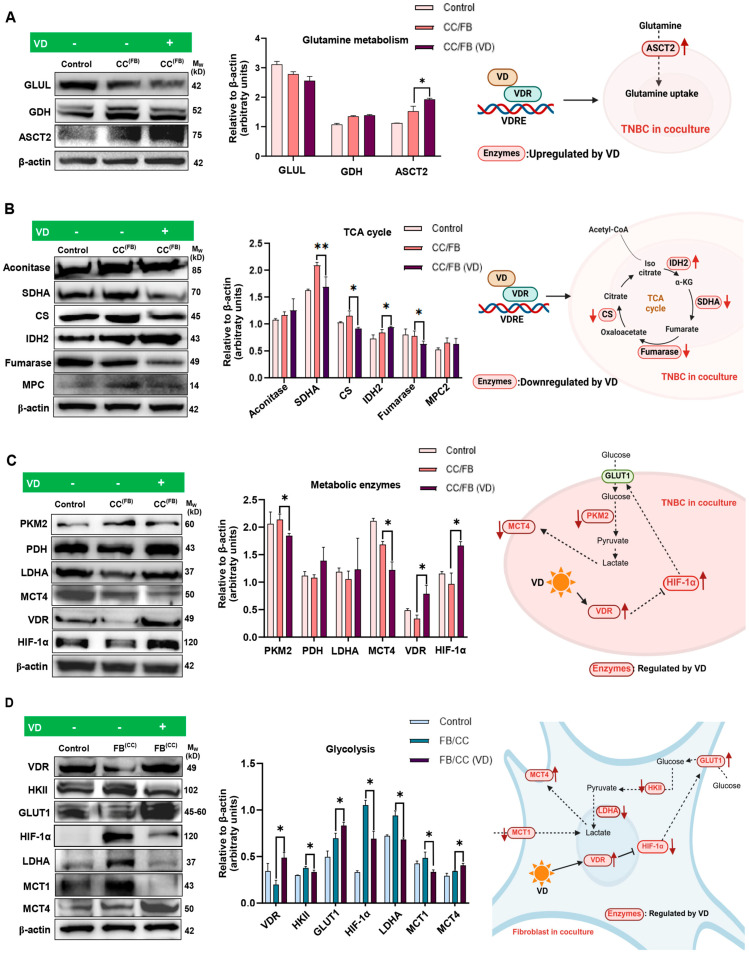
MDA-MB-231 and MRC-5 fibroblast co-cultures were treated with 20 nM of VD for 72 h and cancer cells assessed via Western blot for expression of (**A**) glutamine metabolic enzymes (GLUL, GDH, and ASCT2); (**B**) TCA cycle enzymes (Aconitase, SDHA, CS, IDH2, fumarase, and MPC2); (**C**) other metabolic enzymes (PKM2, PDH, LDHA, MCT4, and VDR) and master metabolic regulator, HIF-1α. (**D**) Fibroblasts were also examined for expression of metabolic enzymes (VDR, HKII, GLUT1, LDHA, MCT1, and MCT4) and master metabolic regulator, HIF-1α, following co-culture. (**A**–**D**) Results are represented as mean ± SD (*n* = 3). Significance relative to control cells: * *p* < 0.05, ** *p* < 0.01. The uncropped original Wester blotting images can be found in File S1.

Evaluating glutamine metabolic enzymes in cancer cells, VD treatment only significantly elevated the expression of ASCT2 in the co-cultured cancer cells, indicating increased glutamine uptake by MDA-MB-231 cells in the presence of VD (Figure 2A). Examining the TCA cycle enzymes, VD treatment significantly down-regulated key enzymes such as SDHA, CS, and fumarase in cancer cells (Figure 2B). However, it did not alter the expression of aconitase or MPC2, while notably up-regulating IDH2 levels (Figure 2B). Overall, these findings suggest that VD modulates TCA cycle-associated enzymes in a selective manner, reflecting complex and enzyme-specific effects on TCA cycle activity.

Incubation with VD also significantly down-regulated PKM2, while the levels of PDH and LDHA remained unaffected in cancer cells. Additionally, VD reduced the expression of MCT4 in the co-cultured cancer cells. Notably, expression of the vitamin D receptor (VDR) and HIF-1α were significantly elevated in the co-cultured cancer cells (Figure 2C). Collectively, these observations reflect that VD induces selective and context-dependent metabolic changes in cancer cells under co-culture conditions. The concurrent down-regulation of specific metabolic enzymes alongside enhanced HIF-1α and ASCT2 expression highlights a complex and nuanced metabolic response to VD within the co-culture microenvironment.

Evaluating the effect of VD on fibroblasts, we observed a significant increase in VDR expression, suggesting these cells are also responsive to the effects of VD. However, in contrast to the effects of VD in cancer cells, VD significantly reduced the master metabolic regulator HIF-1α in fibroblasts (Figure 2D). Other metabolic enzymes significantly reduced in the fibroblasts following supplementation with VD included HKII, LDHA, and MCT1 (Figure 2D), suggesting their impaired ability to undergo glycolysis as well as generate and take up lactate. Notably, the glucose importer GLUT1 and lactate exporter MCT4 were up-regulated in these cells, highlighting a selective and pathway-specific metabolic response rather than a uniform metabolic suppression.

Overall, our findings suggest that VD treatment partially counteracts cancer-driven metabolic re-programming of fibroblasts in co-culture by selectively modulating glycolytic and lactate processing pathways.

### 3.5. VE Up-Regulates the TCA Cycle Activity in MDA-MB-231 Cells Co-Cultured with Fibroblasts

**Figure 3:**
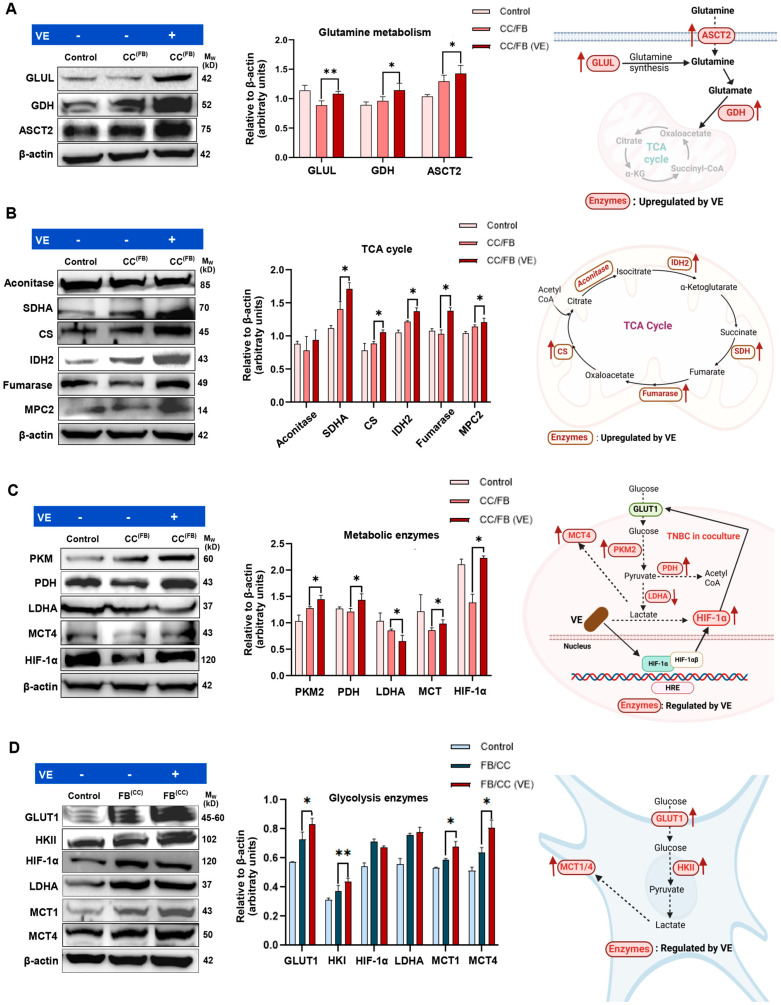
MDA-MB-231 and MRC-5 fibroblasts co-cultures were treated with 20 µM of VE for 72 h and cancer cells assessed via Western blot for expression of (**A**) glutamine metabolic enzymes (GLUL, GDH, and ASCT2); (**B**) TCA cycle enzymes (Aconitase, SDHA, CS, IDH2, fumarase, and MPC2); (**C**) other metabolic enzymes (PKM2, PDH, LDHA, and MCT4) and master metabolic regulator, HIF-1α. (**D**) Fibroblasts were also examined for metabolic enzymes (GLUT1, HKII, LDHA, MCT1, and MCT4) and master metabolic regulator, HIF-1α, following co-culture. (**A**–**D**) Results are represented as mean ± SD (*n* = 3). Significance relative to control cells: * *p* < 0.05, ** *p* < 0.01. The uncropped original Wester blotting images can be found in File S1.

We next examined whether VE influences the metabolic crosstalk between MDA-MB-231 cells and MRC-5 fibroblasts. Evaluating the effect of VE on MDA-MB-231 cancer cells following co-culture with fibroblasts, we observed a significant increase in the expression of GLUL, GDH, and ASCT2 (Figure 3A), indicating altered regulation of glutamine uptake and metabolism. Further, the TCA cycle enzymes, including SDHA, CS, IDH2, and Fumarase were all significantly up-regulated in cancer cells following exposure to VE (Figure 3B). MPC2, which shuttles pyruvate into mitochondria, was also markedly enhanced by VE (Figure 3B). Collectively, these observations indicate that VE treatment is associated with increased expression of glutamine and mitochondria-associated metabolic enzymes in cancer cells under co-culture conditions.

The glycolysis enzyme, PKM2, which facilitates pyruvate production, and mitochondrial enzyme, PDH, which converts pyruvate to Acetyl-CoA, were both also elevated in response to VE, which further supports more pyruvate being fed into the TCA cycle (Figure 3C). Notably, LDHA was reduced by VE suggesting less pyruvate was being converted to lactate, which is consistent with its increased uptake into the mitochondria. MCT4, which exports lactate, was up-regulated in the cancer cells following VE treatment (Figure 3C), although the effect of this is unlikely to be significant as lactate production in these cells was reduced. Finally, HIF-1α was increased in the cancer cells following VE treatment, an effect that was similar to that observed with VD treatment. The simultaneous increase in HIF-1α and TCA cycle-associated enzymes may indicate an adaptive metabolic response to the co-culture microenvironment, highlighting the complexity of metabolic regulation in response to VE treatment.

Evaluating the effect of VE on fibroblasts co-cultured with MDA-MB-231 cells, we found that VE significantly up-regulated the expression levels of GLUT1 and HKII, suggesting increased glucose uptake and metabolism by fibroblasts. The lactate transporters MCT1 and MCT4 were also both up-regulated in VE-treated fibroblast co-cultures. However, the expression levels of HIF-1α and LDHA remained unchanged in fibroblasts (Figure 3D).

Overall, our findings suggest that VE enhances glutamine metabolism capacity alongside TCA cycle enzymes in co-cultured cancer cells, while also increasing glycolysis enzymes in co-cultured fibroblasts. This suggests that unlike VD, VE may potentially sustain metabolic crosstalk between BrCa cancer cells and fibroblasts under the co-culture conditions.

## 4. Discussion

**Figure 4:**
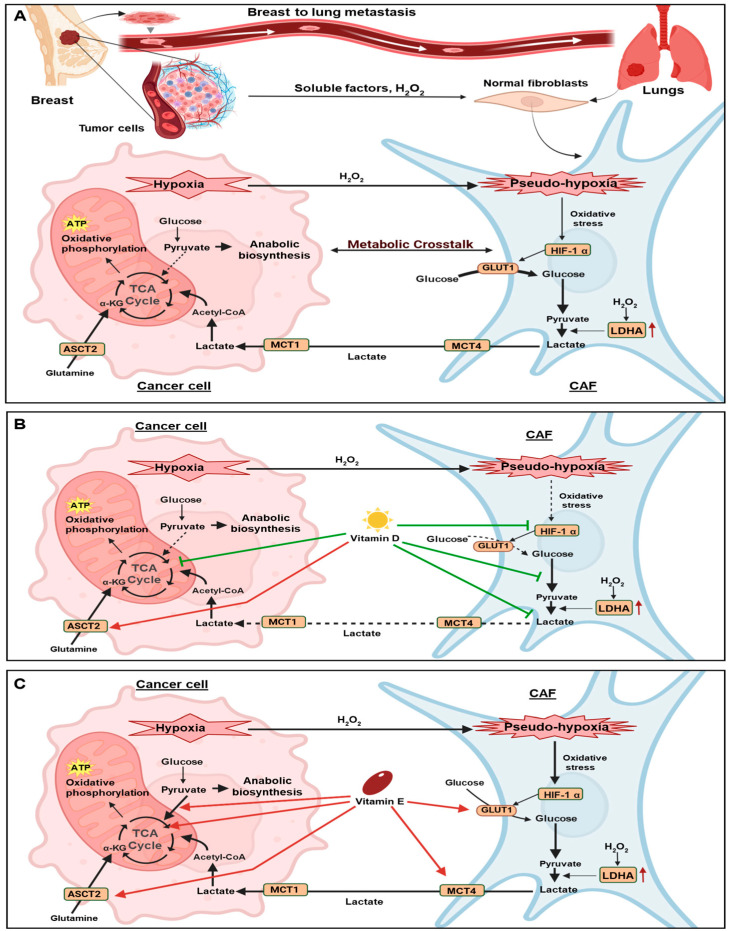
Proposed model of metabolic crosstalk between BrCa cells and lung fibroblasts. This schematic represents the proposed metabolic interactions between MDA-MB-231 cells and MRC-5 lung fibroblasts in co-culture. (**A**) TNBC cells release signalling molecules that activate fibroblasts into CAFs, promoting metabolic crosstalk that supports cancer progression. (**B**) Vitamin D potentially enhances glutamine uptake enzymes while reducing TCA cycle enzymes in cancer cells and suppresses lactate metabolism and HIF-1α expression in fibroblasts. (**C**) Vitamin E potentially increases glutamine metabolism and OXPHOS dependence in cancer cells while potentially promoting glucose uptake and lactate export in fibroblasts within the co-culture system.

In this pilot study, we used an indirect co-culture system to investigate the interactions between MDA-MB-231 TNBC cells and MRC-5 lung fibroblasts to recapture the TME during metastasis. This co-culture system revealed increased expression of α-SMA and FAP in MRC-5 fibroblasts, indicating their transformation into CAFs. The activation of lung fibroblasts into CAFs, which are critical components of the tumour stroma is mediated by cancer cell-derived exosomes and cytokines (Figure 4A) [39]. Once activated, CAFs secrete cytokines, chemokines, metabolites, and growth factors that significantly contribute to cancer metastasis [40,41].

Our results also revealed distinct metabolic changes in both cancer cells and fibroblasts within the co-culture system. The MDA-MB-231 cancer cells exhibited increased expression of TCA cycle-associated enzymes when co-cultured with fibroblasts, suggesting a possible increase in metabolic activity, which may be fueled by glutamine uptake as well as CAF-derived metabolites (Figure 4A). In parallel, glycolytic enzymes in co-cultured fibroblasts were up-regulated, which may suggest increased lactate production and export. This lactate could possibly contribute to metabolic crosstalk with cancer cells to support mitochondrial metabolism and anabolic processes (Figure 4A) [42], although further direct assessment of lactate transfer between these cells is required. Notably, studies have shown that increased lactate export contributes to extracellular acidification in the TME, a phenomenon that impairs T-cell function and promotes immunosuppression [43]. These findings suggest that metabolic crosstalk between cancer cells and fibroblasts not only supports tumour progression but also influences immune regulation within the TME.

Notably, while most TCA cycle enzymes were up-regulated in co-cultured MDA-MB-231 cells, fumarase expression was reduced. Further, its expression was increased in co-cultured fibroblasts. This opposing regulation of fumarase may reflect active cell-type-specific metabolic re-programming. Previous studies have reported that fumarase expression can be transcriptionally and epigenetically regulated by chromatin-remodelling factors such as lymphocyte-specific helicase (LSH), leading to its suppression in certain cancers [44]. Furthermore, fumarase has been reported to possess non-metabolic functions, including roles in DNA damage response pathways, which may influence its expression and subcellular localisation in response to microenvironment-associated stress [45]. It has also been reported that a reduction in fumarase activity leads to accumulation of fumarate, which acts as an oncometabolite by driving epigenetic changes that promote the epithelial-to-mesenchymal transition (EMT), ultimately promoting tumour invasion and metastasis [46]. In line with these findings, the decreased levels of fumarase observed in our study indicates a metabolic adaptation in TNBC cells, which may contribute to tumour progression within the co-culture context, although whether this is linked to EMT requires further examination.

We further demonstrate that pyruvate is likely being diverted away from the TCA cycle in cancer cells following co-culture. This was evident from the reduced levels of PDH, the enzyme that converts pyruvate into Acetyl-CoA for entry into the TCA cycle. The reduced levels of LHDA in cancer cells further suggest pyruvate is not being used to generate lactate. Hence, it is plausible that the pyruvate generated by cancer cells is potentially being used to fuel other anabolic processes. This metabolic adaptation aligns with previous reports indicating that pyruvate serves as a precursor for lipogenesis, amino acid biosynthesis, gluconeogenesis, and nucleotide synthesis (46). The increased expression of glutamine transporter ASCT2 and GDH, which shuttles glutamine into the TCA cycle by converting it to α-KG, further suggests that TNBC cells in co-culture derive a significant portion of their energy from extracellular glutamine, which replenishes the TCA cycle. This metabolic re-programming enhances cancer cell fitness by supporting biosynthetic and proliferative demands.

The mechanisms responsible for the observed metabolic re-programming are likely mediated by HIF-1α, which plays a critical role in metastasis [36] and is a master regulator of metabolism [14]. In our study, HIF-1α was down-regulated in cancer cells but up-regulated in fibroblasts in the co-culture system. This differential expression reflects the distinct metabolic adaptations of cancer cells and fibroblasts in the TME. Notably, a study demonstrated that elevated levels of reactive oxygen species (ROS) originating in cancer cells can induce oxidative stress in neighbouring CAFs, leading to the activation and stabilisation of HIF-1α [22]. Although HIF—1α expression was reduced in TNBC cells during co-culture, the observed increase in TCA cycle enzyme expression may reflect a shift toward glutamine-dependent metabolism, supported by increased glutamine uptake via ASCT2. This metabolic adaptation is likely driven by stromal interactions in the co-culture environment and occurs independently of HIF—1α. HIF—1α-independent metabolic re-programming in the context of cancer-stroma crosstalk has been previously reported, supporting the role of the TME in shaping cancer cell metabolism [47]. HIF-1α was previously found to promote glycolysis under hypoxic conditions [22,48] and may be the driving force behind the increased glycolysis observed in the CAFs following co-culture with cancer cells. Notably, HIF-1α was found to facilitate the formation of a pre-metastatic niche in the lungs through the lysyl oxidase (LOX) family, further supporting its role in metastatic progression [49] and highlighting its important role in metabolic crosstalk within the BrCa TME.

Examining how supplementation of the co-culture media with either VD or VE influences metabolic crosstalk between BrCa cells and fibroblasts, we observed some notable differences. VD has a well-established role in inhibiting BrCa metastasis [50], with most studies reporting that VD down-regulates HIF-1α [51,52]. In the present study, VD was evaluated across a range of physiologically relevant and supraphysiological (pharmacological) concentrations (10–100 nM) [32,53], identifying 20 nM as an effective dose that was used for subsequent experiments. VD primarily exerts its effects via the VDR, which has a crucial role in regulating tumour metabolism by influencing mitochondrial function, OXPHOS, and biosynthetic pathways [54,55]. Furthermore, VDR signalling was shown to attenuate proliferation, induce apoptosis and reduce migration/invasion in TNBC [55,56]. Our study demonstrates that co-culture led to down-regulation of VDR expression in both cancer cells and fibroblasts, suggesting that interactions within the TME suppress VDR signalling. However, VD treatment reversed this, restoring VDR expression in both cell types.

Interestingly, Brożyna et al. demonstrated a negative correlation between VDR and HIF-1α expression in primary and advanced metastatic lesions, with highest HIF-1α expression observed in VDR-negative melanoma. Given that HIF-1α regulates metabolic adaptation in tumours, its inverse association with VDR suggests that hypoxia-driven HIF-1α signalling may actively suppress VDR [57]. We observed that in TNBC cells, both VDR and HIF-1α are enhanced following VD treatment, whereas in fibroblasts, VD treatment up-regulated VDR but suppressed HIF-1α expression. This was accompanied by reduced glycolysis in fibroblasts, an effect that is likely mediated by HIF-1α down--regulation [58]. The opposing effects of VD on HIF-1α in cancer cells and fibroblasts likely reflect cell-type specific regulatory mechanisms and microenvironmental influences, highlighting complex metabolic crosstalk within the TME, necessitating more exploration of underlying signalling mechanisms of these interactions. Nonetheless, our study is the first to report that VD potentially suppresses glycolysis in co-cultured CAFs, highlighting a novel mechanism by which VD potentially modulates the TME (Figure 4B).

VE also influenced the metabolic behaviour of both cell types in our co-culture system. VE is an antioxidant nutraceutical whose effective concentrations vary across experimental settings. In vitro studies commonly employ VE within a broad micromolar range (approximately 5–80 µM) to evaluate its biological effects in different disease contexts [31]. Following pilot studies examining VE at doses ranging from 5 to 80 µM, we identified 20 µM as an effective dose that was used in the present study. Whether this dose influences the generation of ROS within the co-culture context was not assessed in this study and remains to be established. However, this dose of VE did influence the expression of numerous metabolic enzymes in both cancer cells and fibroblasts. In cancer cells, VE up-regulated enzymes involved in the production of pyruvate and its entry into the TCA cycle, while also up-regulating multiple TCA cycle enzymes. This is in contrast to the study by Dronamraju et al., which reported that VE suppresses glycolysis in MDA-MB-231 cells [59]. In fibroblasts, VE also increased GLUT1 and HKII expression, suggesting increased glycolysis. Although research on the effects of VE on BrCa CAFs is limited, one study highlighted its protective role in pulmonary fibrosis by suppressing the pathological activation of lung fibroblasts through inhibition of α-SMA and EMT in vitro [60]. Another study demonstrated that VE protected human dermal fibroblasts from oxidative stress [61]. Notably, these latter studies did not consider the influence of cancer-fibroblast crosstalk, examining cancer cells or fibroblasts in isolation, which may account for the contrasting results. Overall, our study reveals that VE may potentially enhance the metabolic interplay between MDA-MB-231 cells and fibroblasts (Figure 4C), an effect that requires further investigation to understand its biological significance and potential redox effects in TNBC lung metastases.

## 5. Limitations and Directions for Future Research

While these findings provide valuable insights into metabolic interactions in metastatic BrCa, our study had limitations. First, the in vitro model does not fully recapitulate the complexity of the TME, including the effect of immune cells, extracellular matrix, and systemic factors. Moreover, the study examined a limited number of metabolic enzymes in a single TNBC cell line. To further validate these results, future studies should assess multiple TNBC and fibroblast cell lines and perform unbiassed metabolic analysis that includes untargeted metabolomics to more accurately dissect the metabolic alterations at play. More physiological conditions such as 3D co-culture systems, patient-derived organoids, or in vivo metastatic models, are needed to validate the intricate metabolic dynamics within the TME and how VD and VE influence this.

## 6. Conclusions

Our findings suggest that the metabolic crosstalk between BrCa cells and lung fibroblasts may play a role in shaping a pro-metastatic environment. Notably, VD appeared to inhibit aspects of oncogenic metabolic crosstalk between BrCa cells and CAFs, suggesting a potential suppressive effect on tumour-supportive interactions. However, VE did not alter this interplay in the same way and was associated with coordinated changes in metabolic enzyme expression in both cell types, suggesting a continued metabolic interplay within the co-culture system.

## Data Availability

The original contributions presented in this study are included in the article/supplementary material. Further inquiries can be directed to the corresponding author.

## Supplementary Materials

The following supporting information can be downloaded at: https://www.mdpi.com/article/10.3390/cancers18020294/s1, File S1: The uncropped original Western blotting images.
