# Supplementary material for: Metabolic Crosstalk in Triple-Negative Breast Cancer Lung Metastasis: Differential Effects of Vitamin D and E in a Co-Culture System
Source: Cancers (Basel). 2026 Jan 18;18(2):294. doi: 10.3390/cancers18020294 (PMC12838710; doi:10.3390/cancers18020294)

Western blot images of MDA-MB-231 and MRC5 coculture

Untreated cancer cell and fibroblasts cocultures:

Figure 1A:

α-Sma:

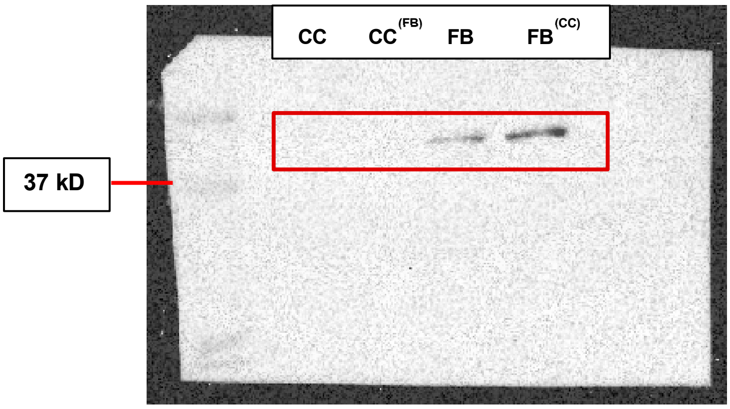

FAP:

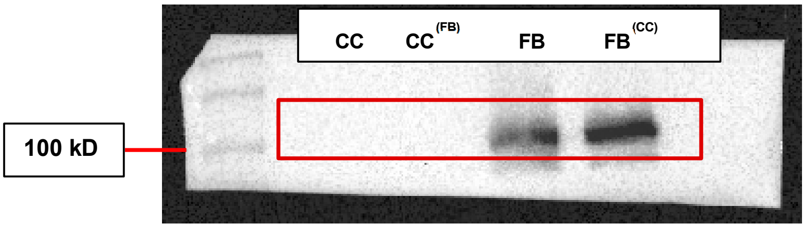

HIF-1 α:

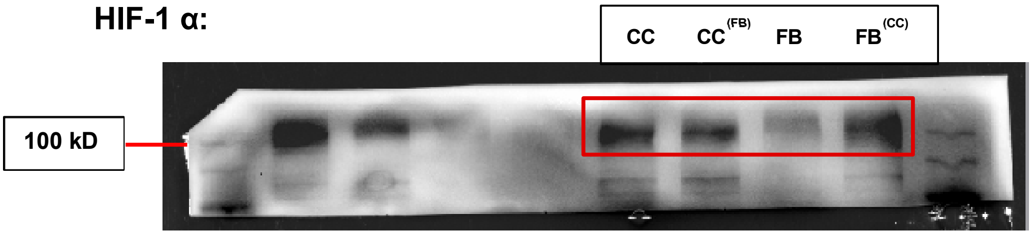

c-Myc:

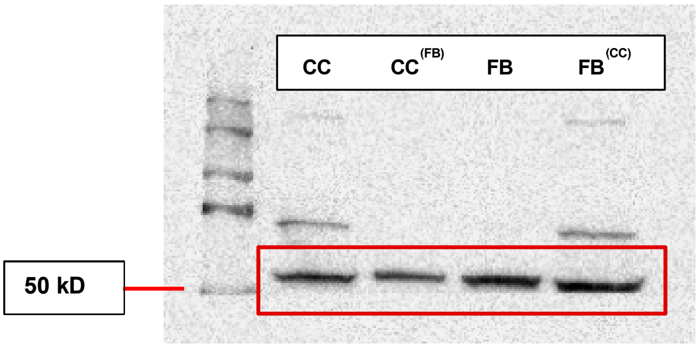

β-actin:

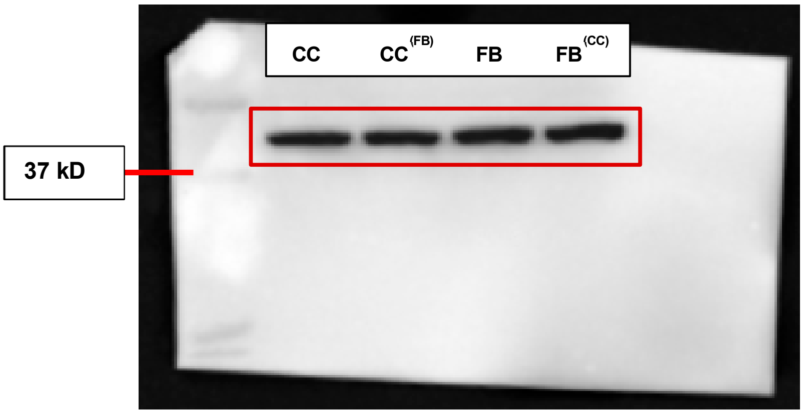

Figure 1D:

GLUL:

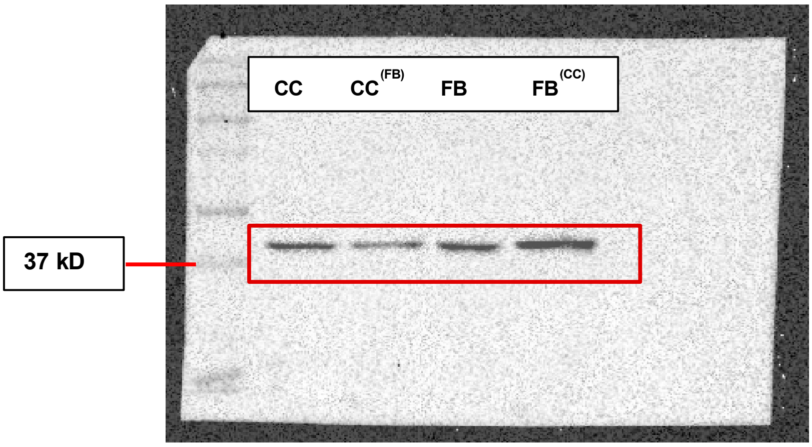

GDH:

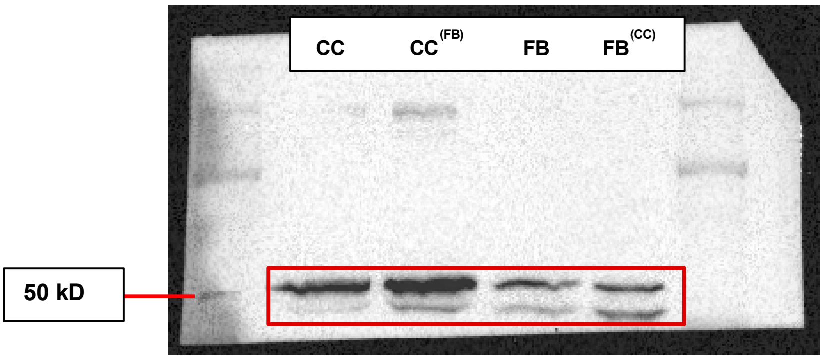

ASCT2:

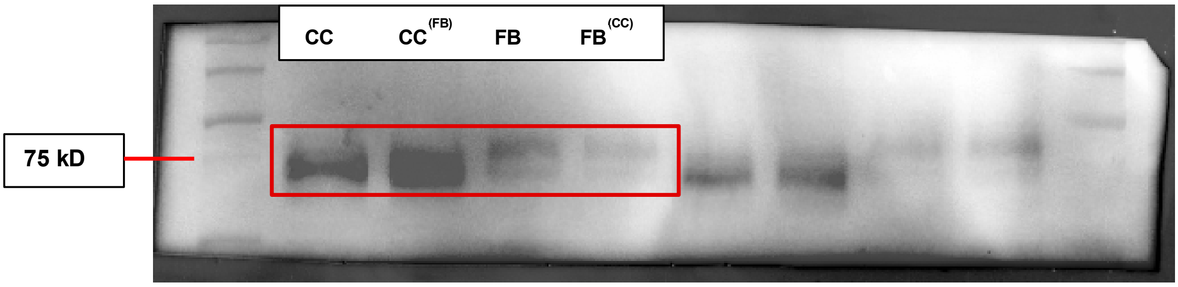

β-actin:

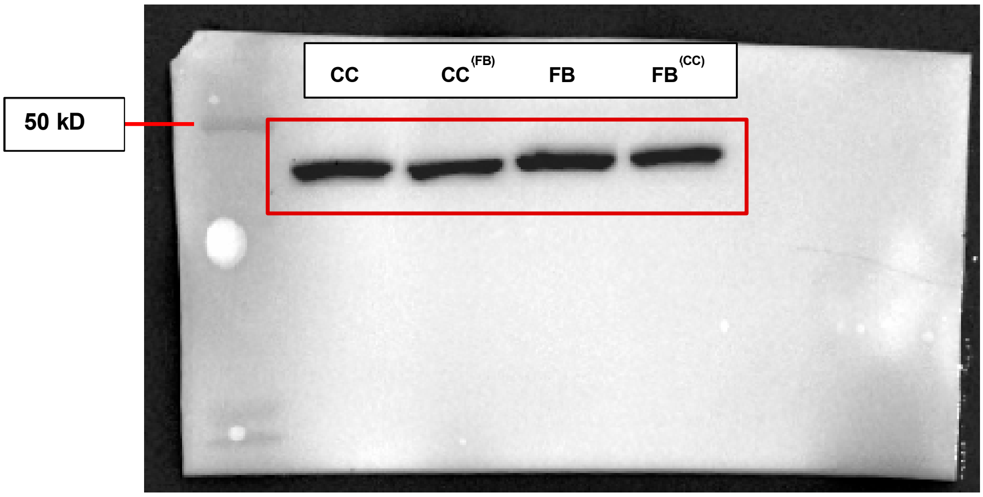

Figure 1E:

Aconitase:

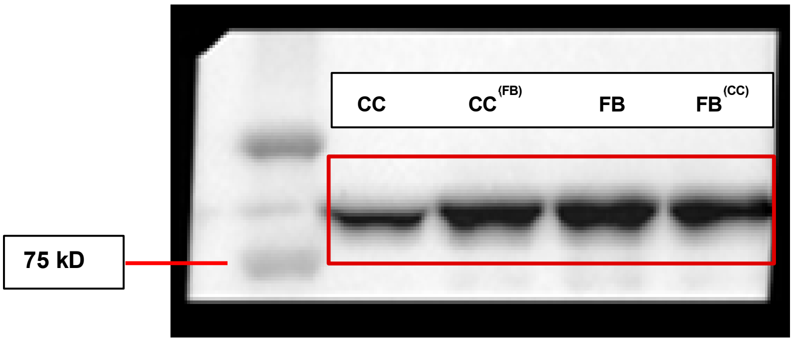

SDHA:

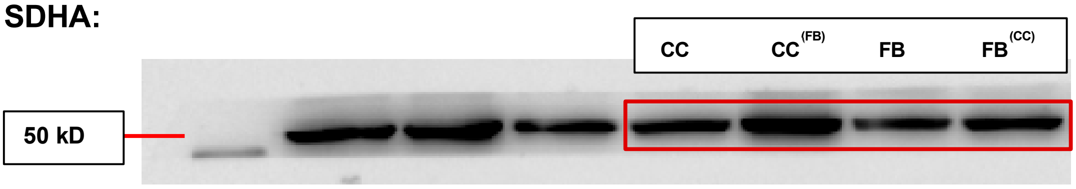

CS:

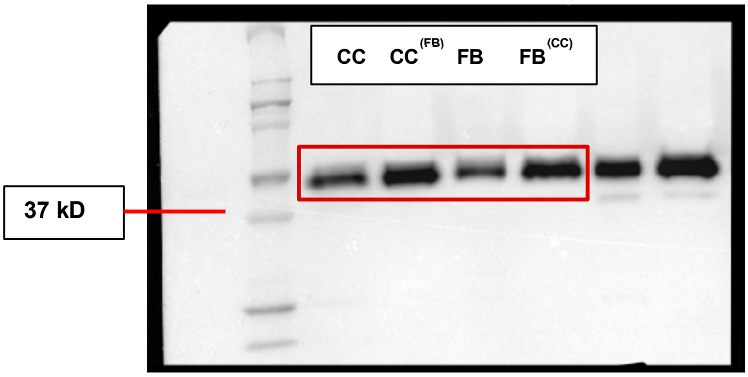

IDH2:

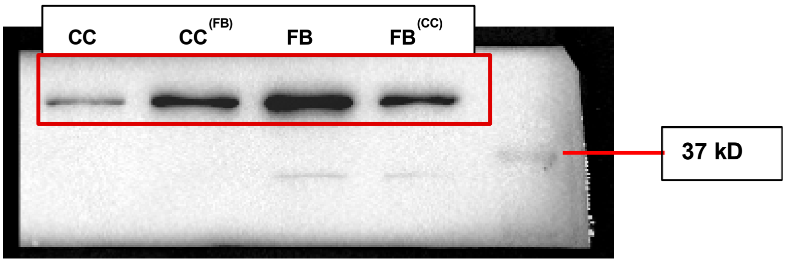

Fumarase:

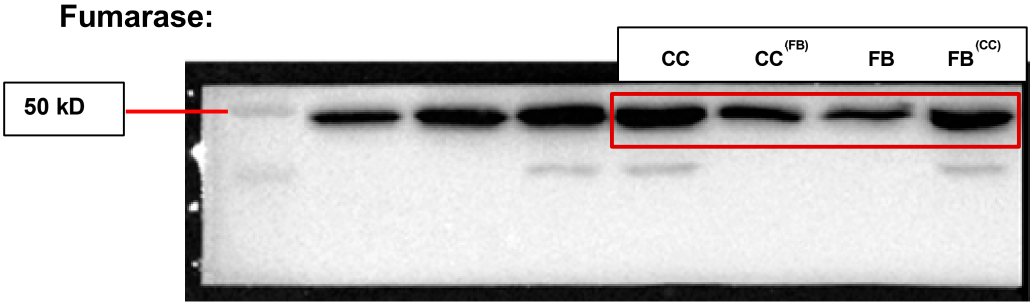

MPC2:

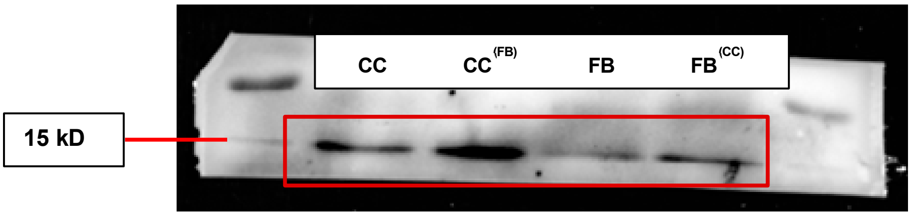

β-actin:

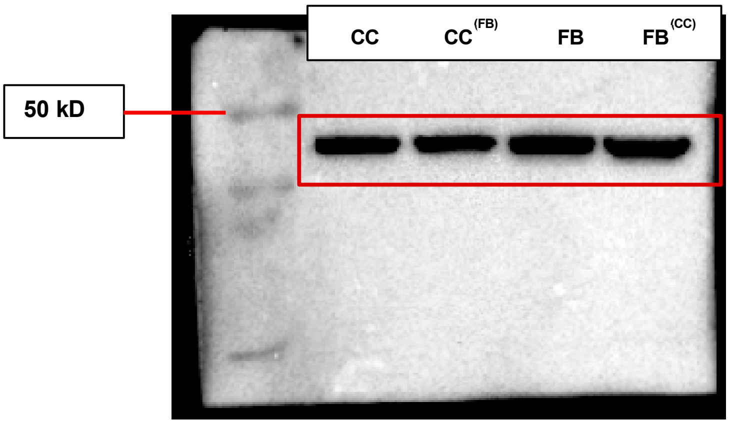

Figure 1F:

GLUT1:

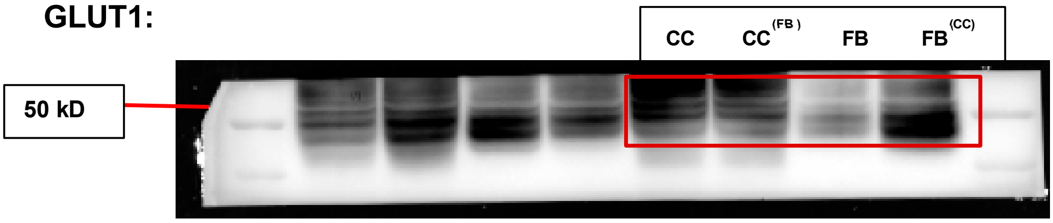

HKII:

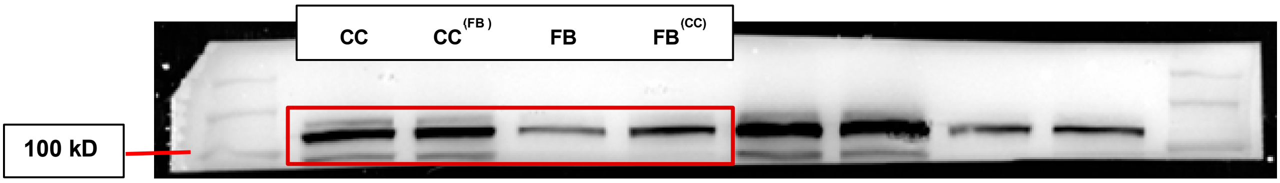

PKM2:

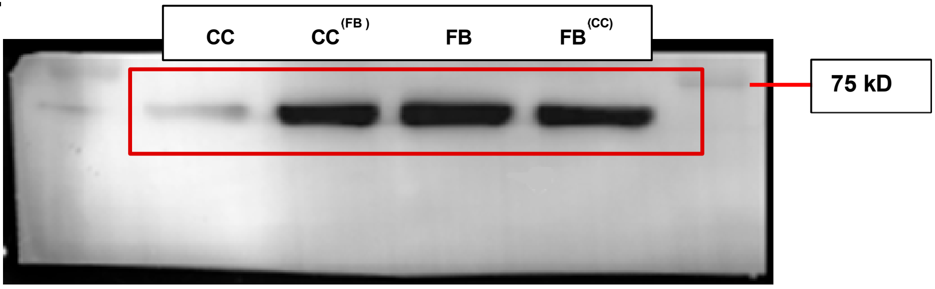

PDH:

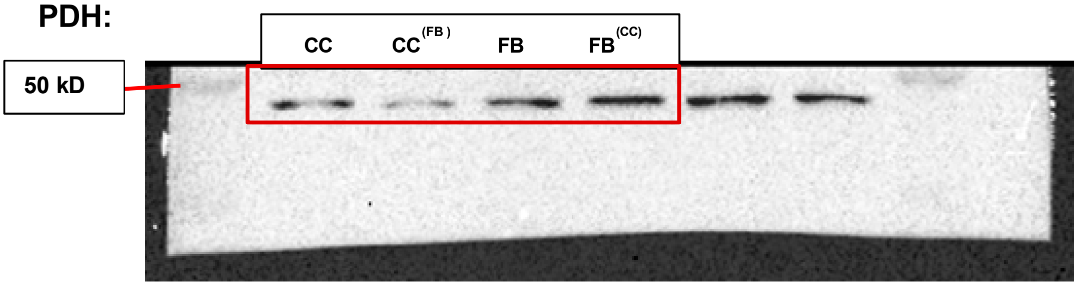

LDHA:

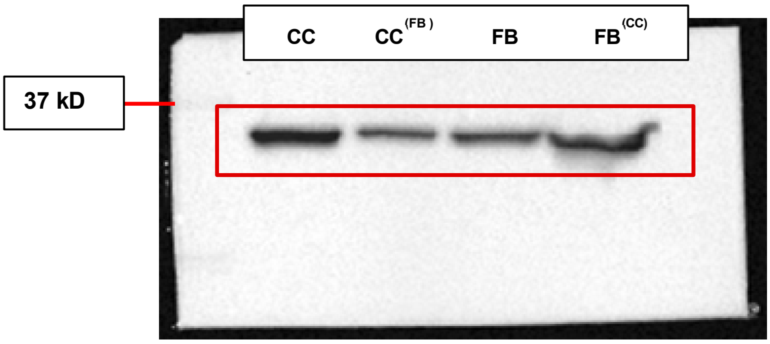

MCT1:

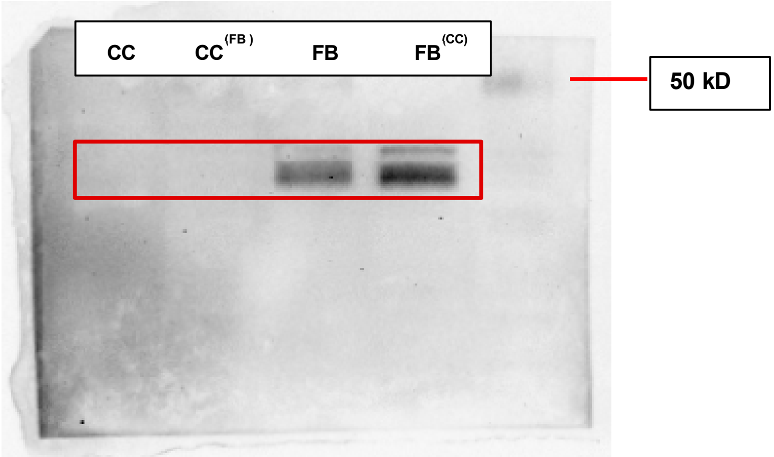

MCT4:

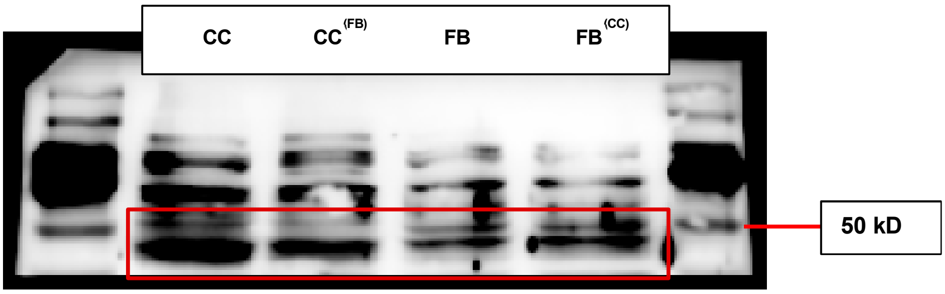

β-actin:

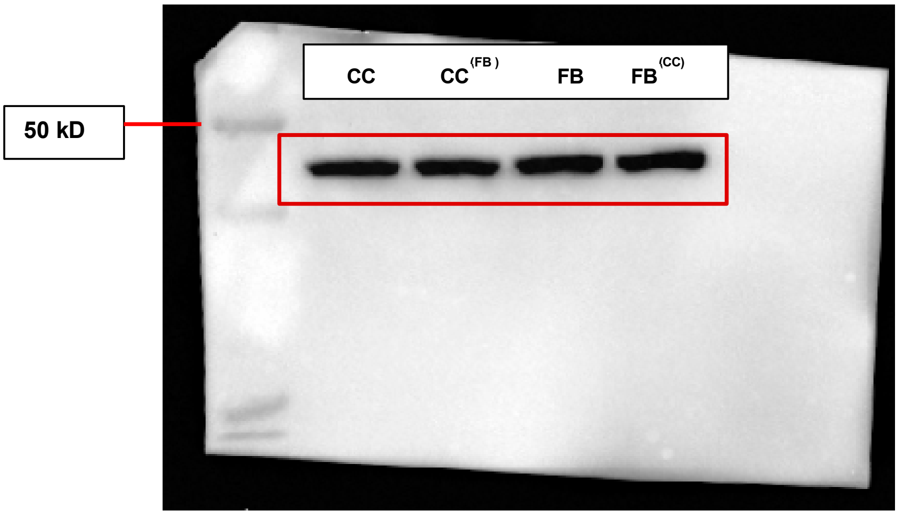

VD treated cocultures:

Figure 2A:

GLUL:

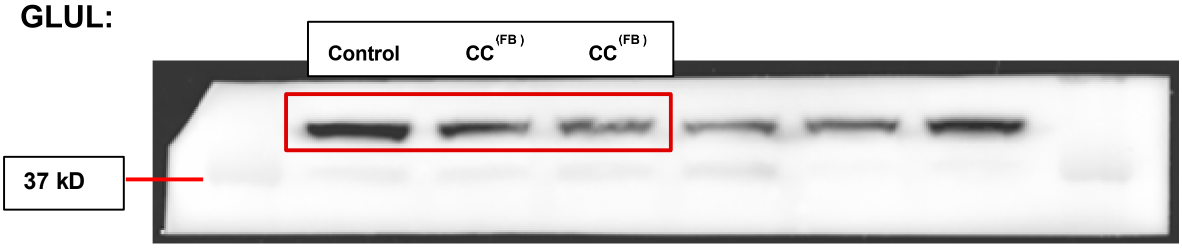

GDH:

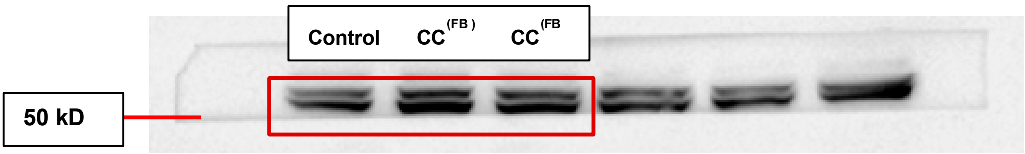

ASCT2:

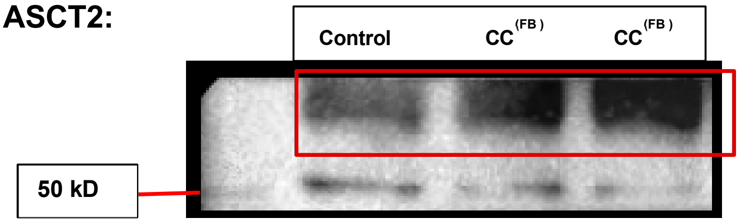

β-actin:

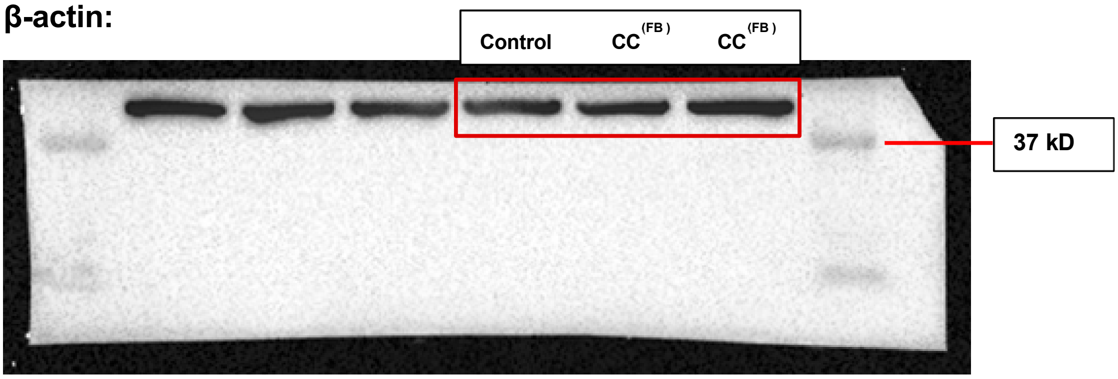

Figure 2B:

Aconitase:

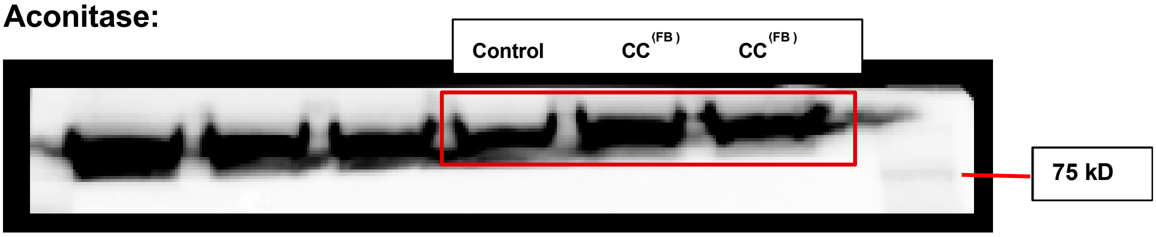

SDHA:

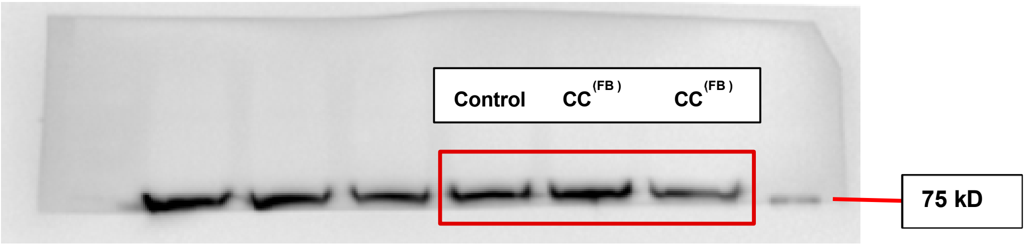

CS:

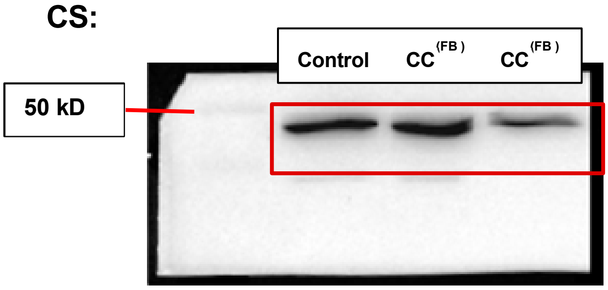

IDH2:

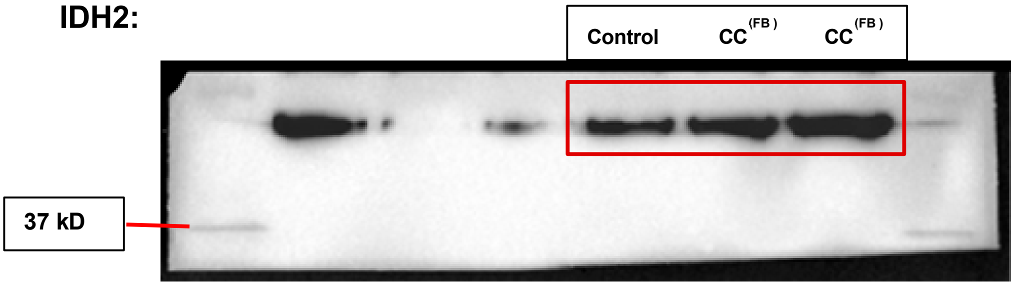

Fumarase:

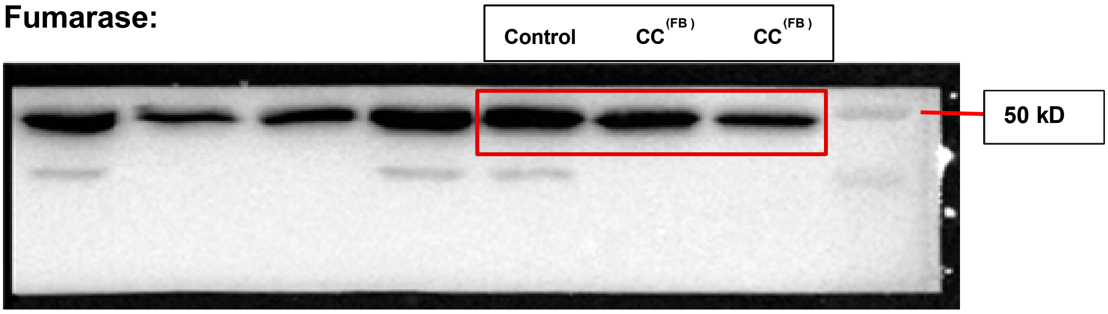

MPC2:

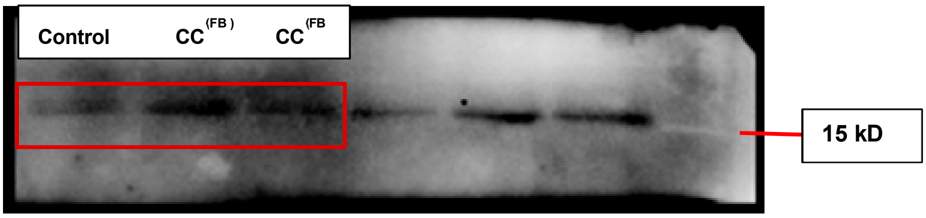

β-actin:

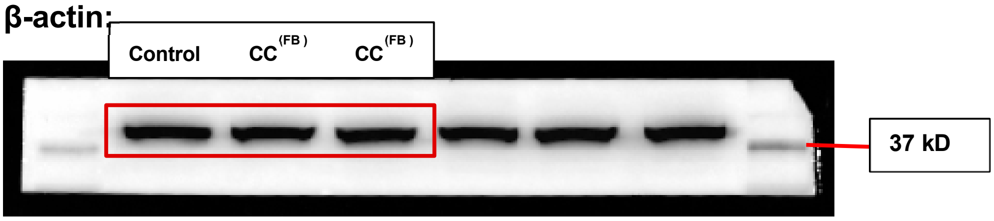

Figure 2C:

PKM2:

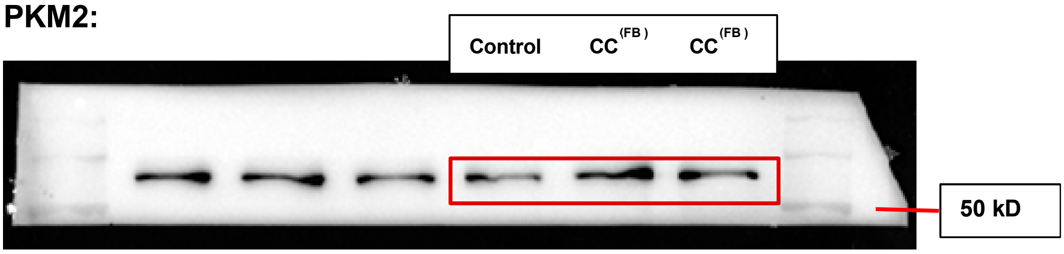

PDH:

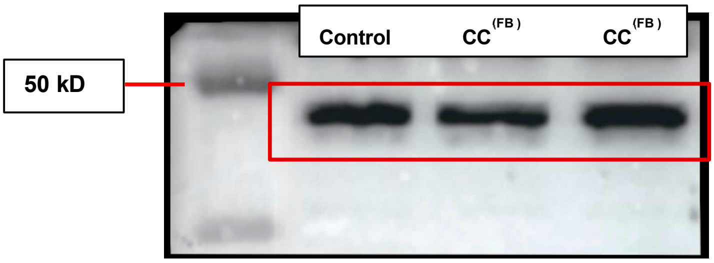

LDHA:

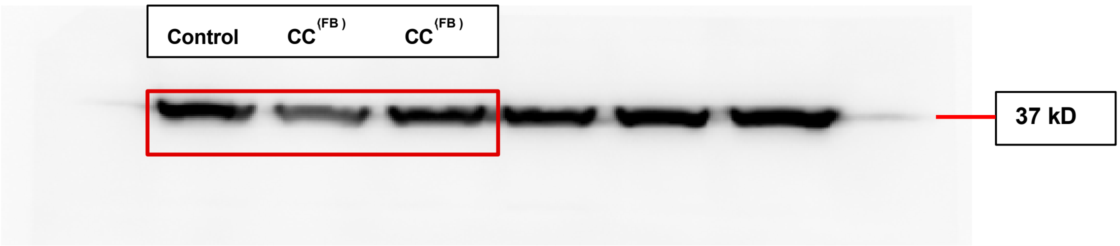

MCT4:

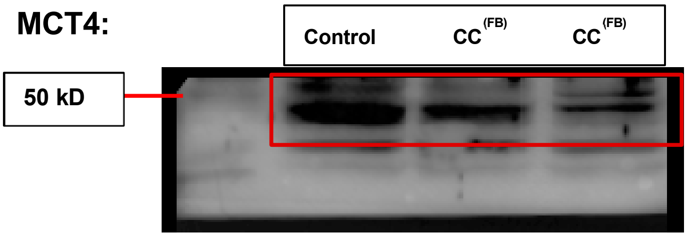

VDR:

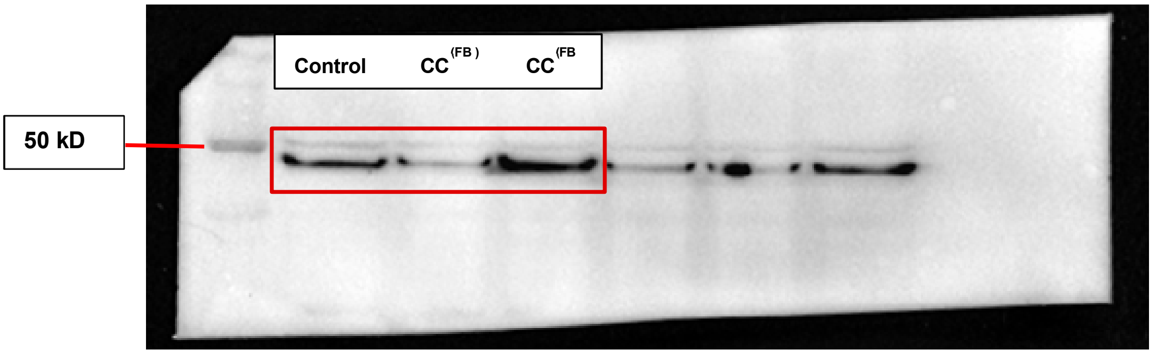

HIF-1 alpha:

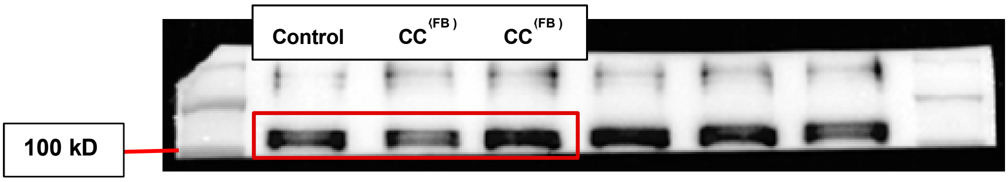

β-actin:

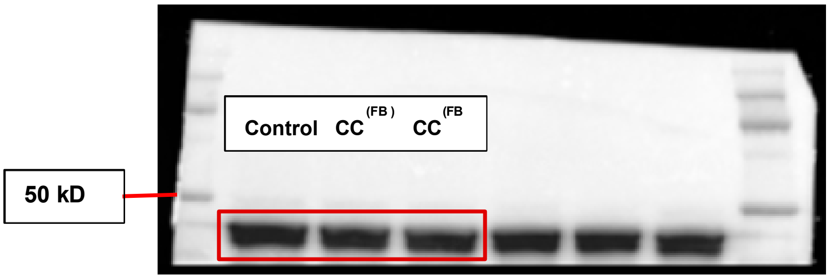

Figure 2D:

VDR:

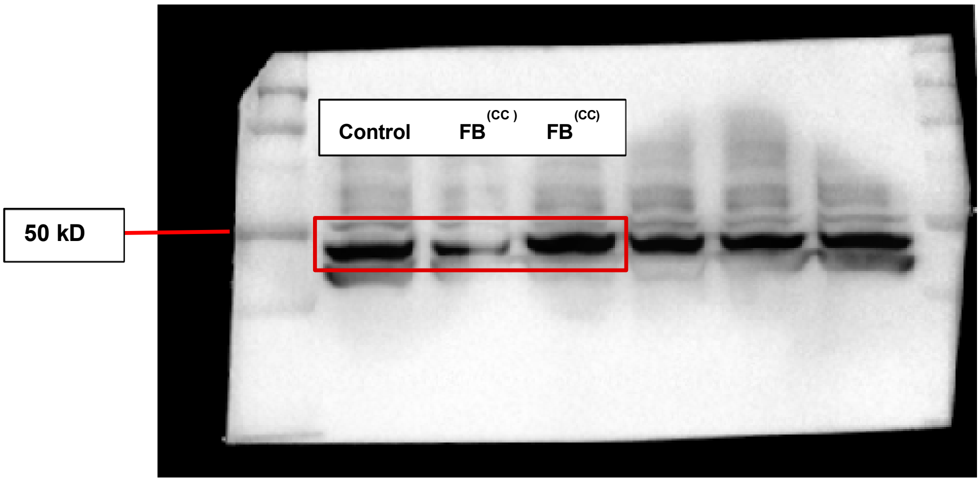

HKII:

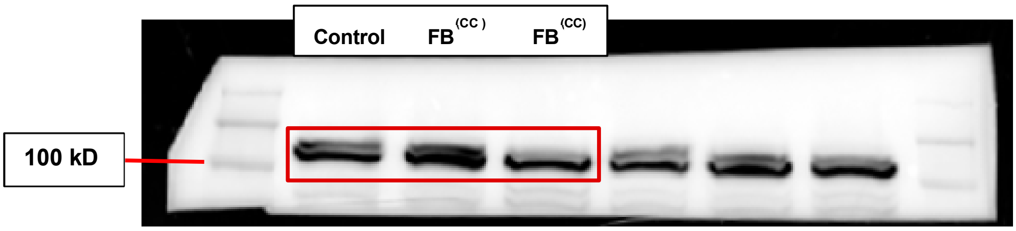

GLUT1:

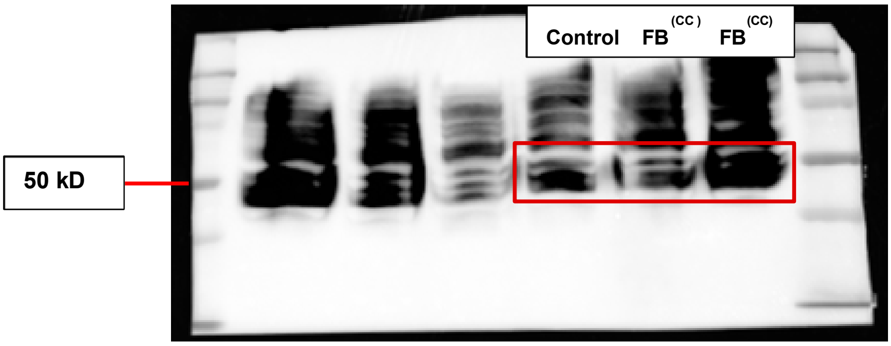

HIF-1 alpha:

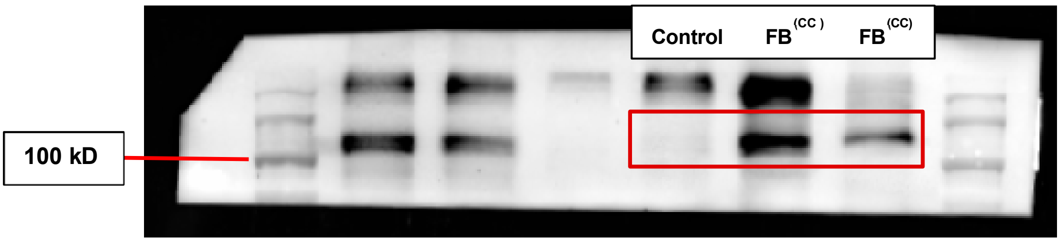

LDHA:

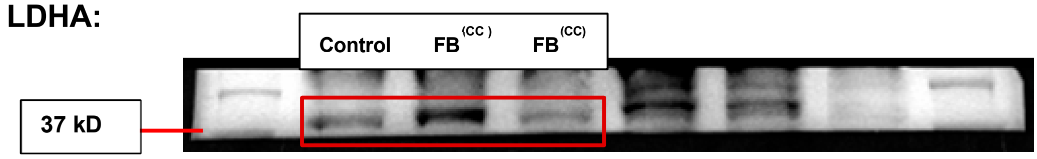

MCT1:

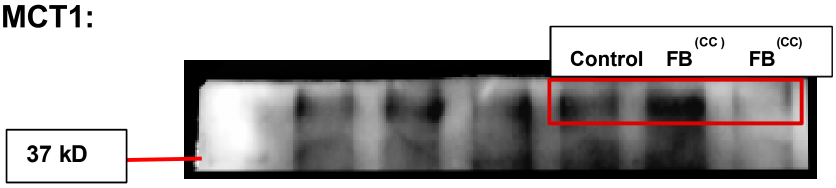

MCT4:

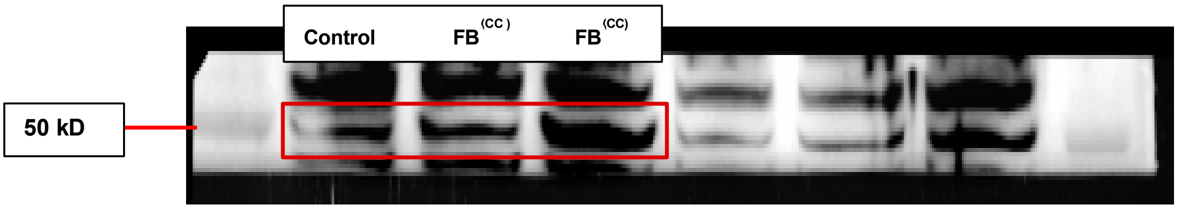

β-actin:

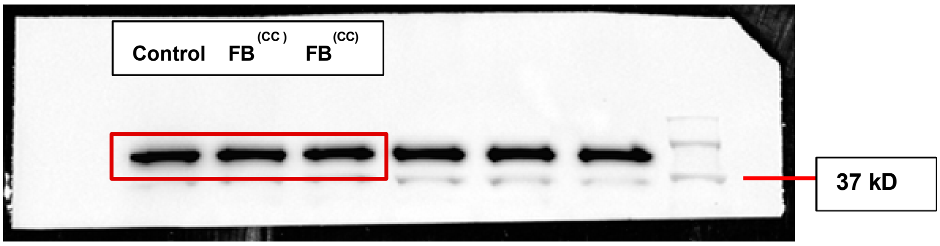

VE treated cocultures:

Figure 3A:

GLUL:

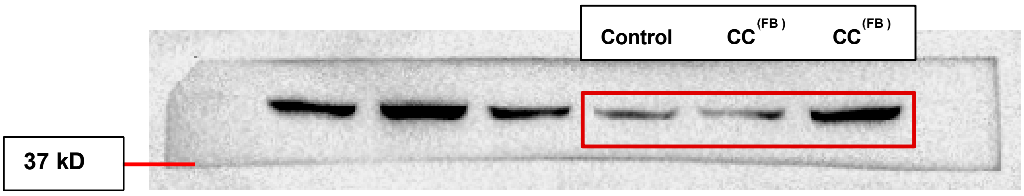

GDH:

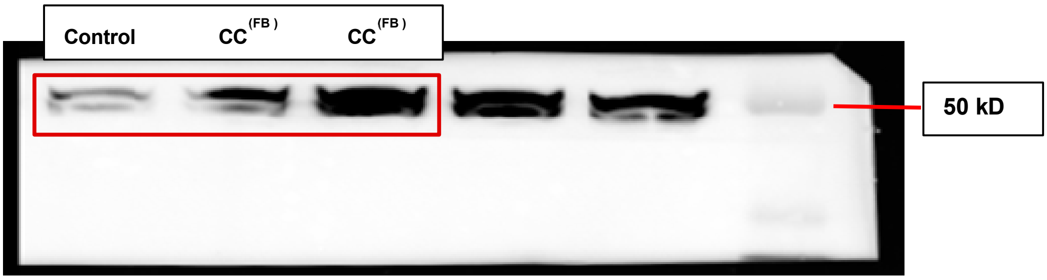

ASCT2:

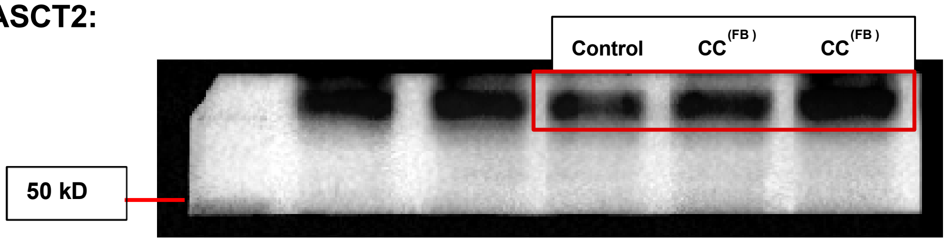

β-actin:

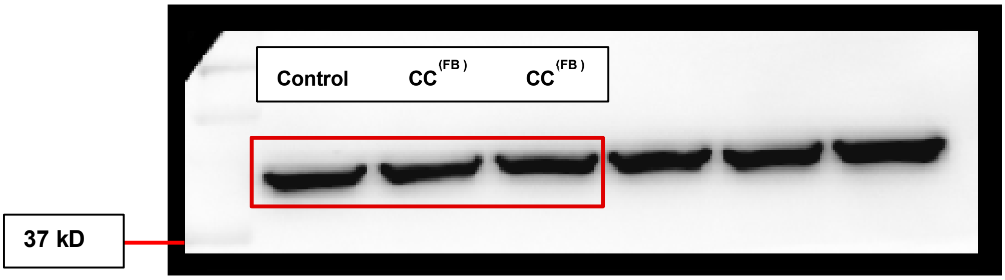

Figure 3B:

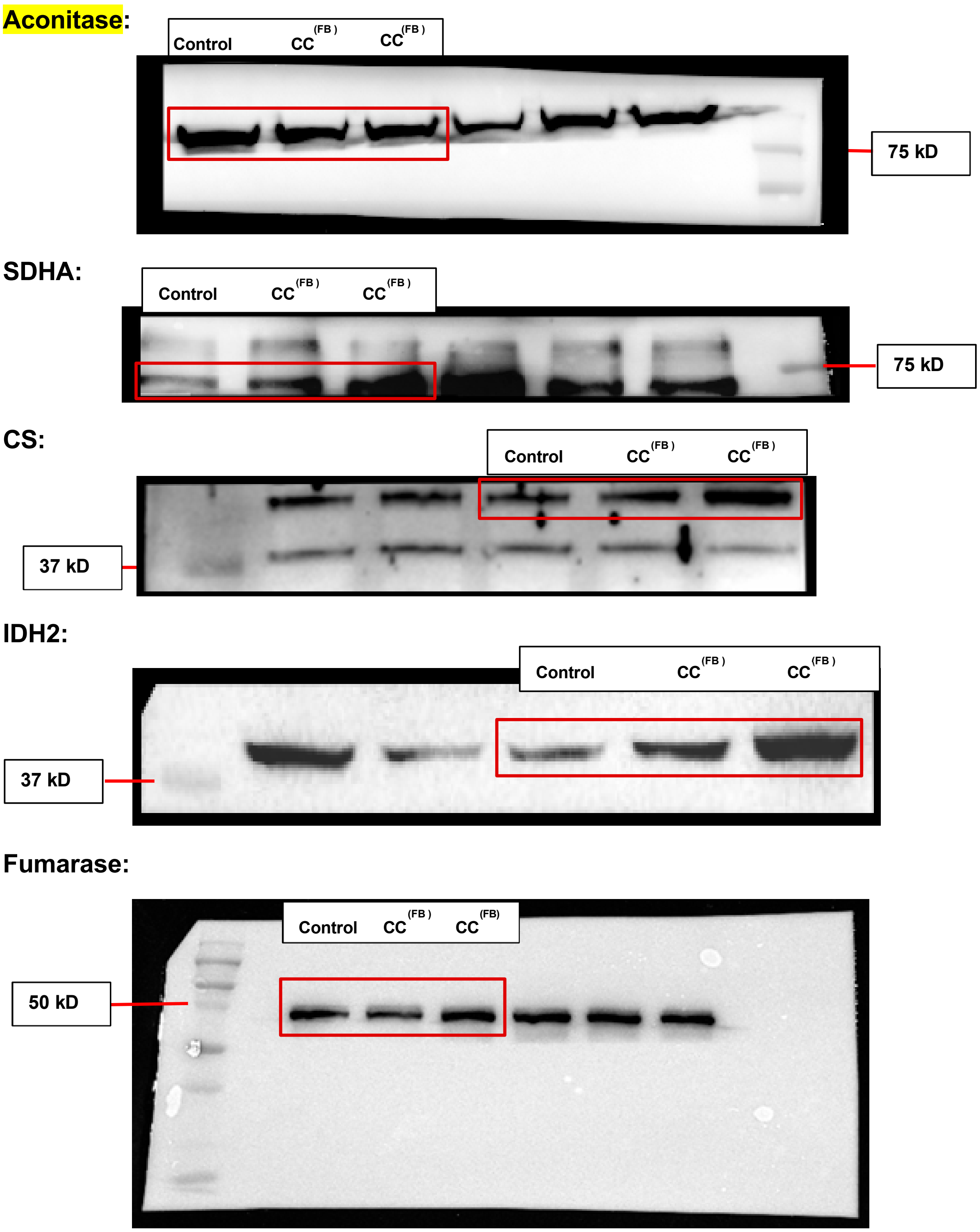

Aconitase:
Control
CC(FB)
CC(FB)
75 kD
SDHA:
Control
CC(FB)
CC(FB)
75 kD
CS:
Control
CC(FB)
CC(FB)
37 kD
IDH2:
Control
CC(FB)
CC(FB)
37 kD
Fumarase:
Control
CC(FB)
CC(FB)
50 kD

MPC2:

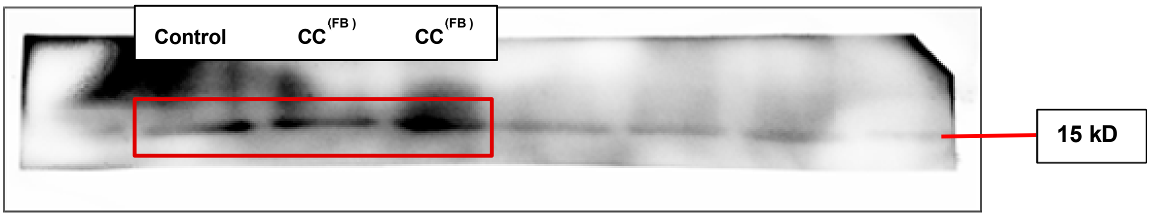

β-actin:

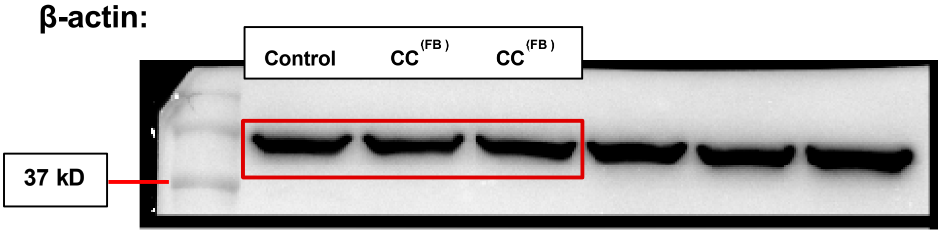

Figure 3C:

PKM2:

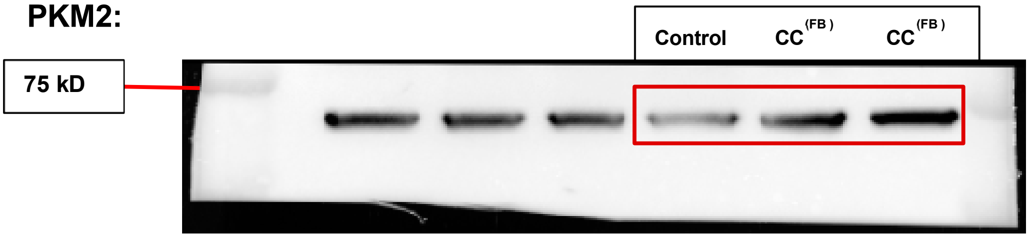

PDH:

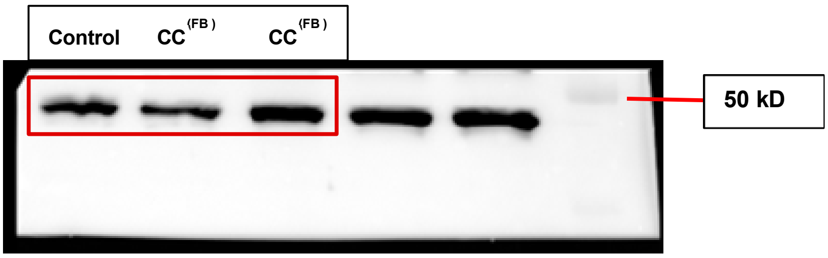

LDHA:

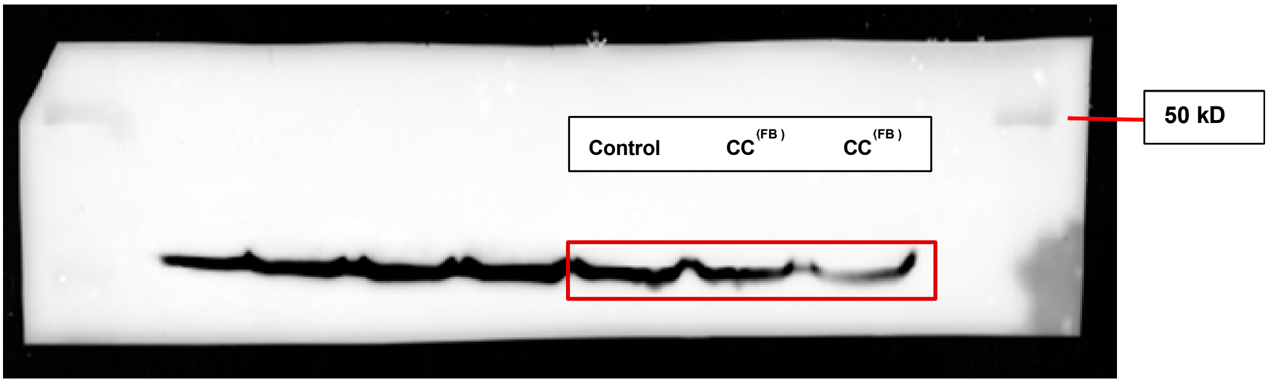

MCT4:

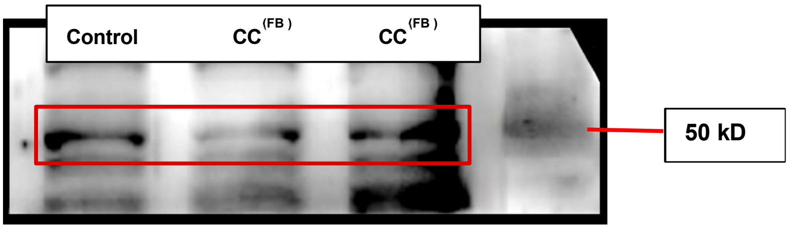

HIF-1 alpha:

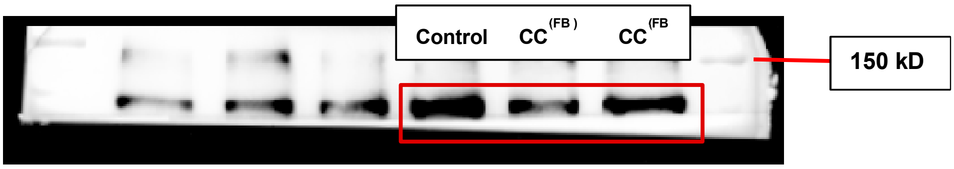

β-actin:

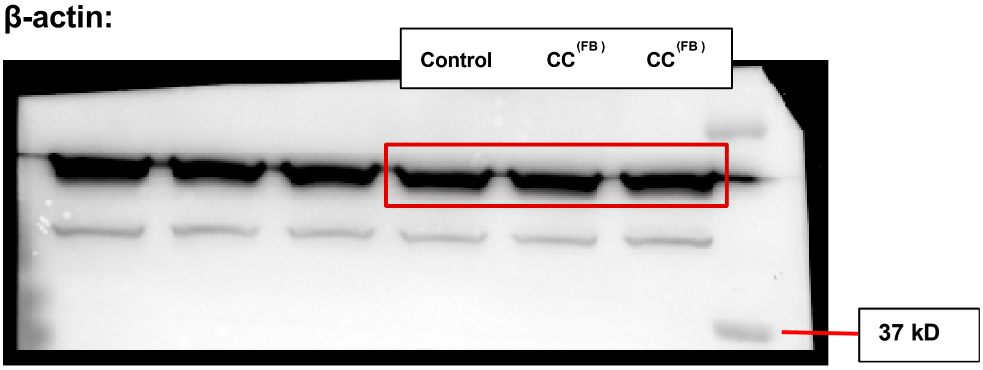

Figure 3D:

GLUT1:

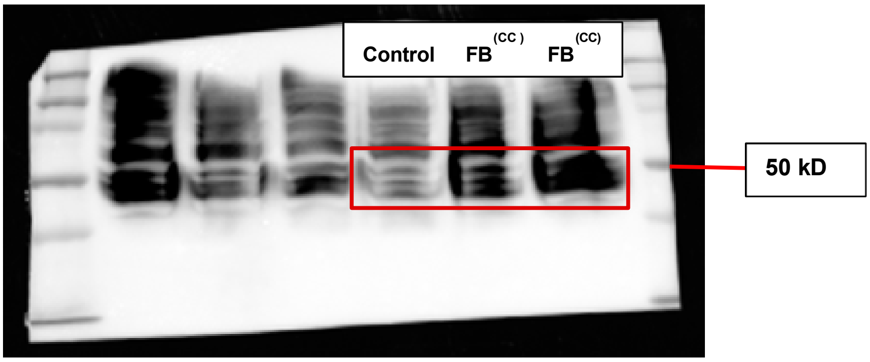

HKII:

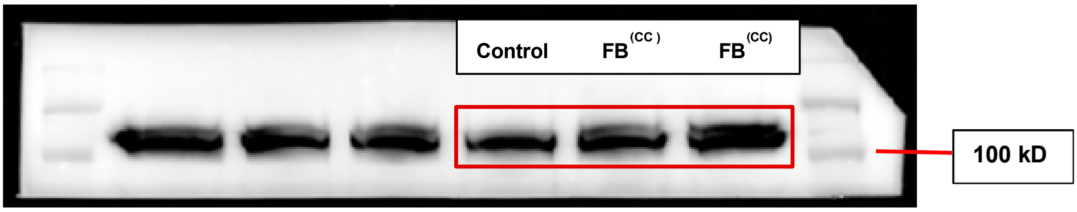

HIF-1 alpha:

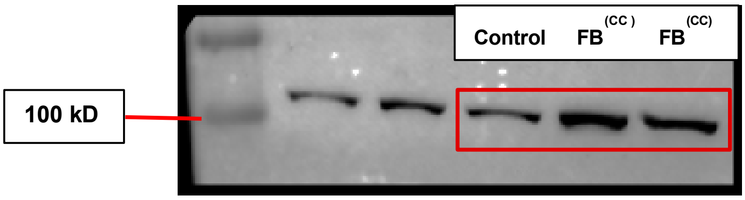

LDHA:

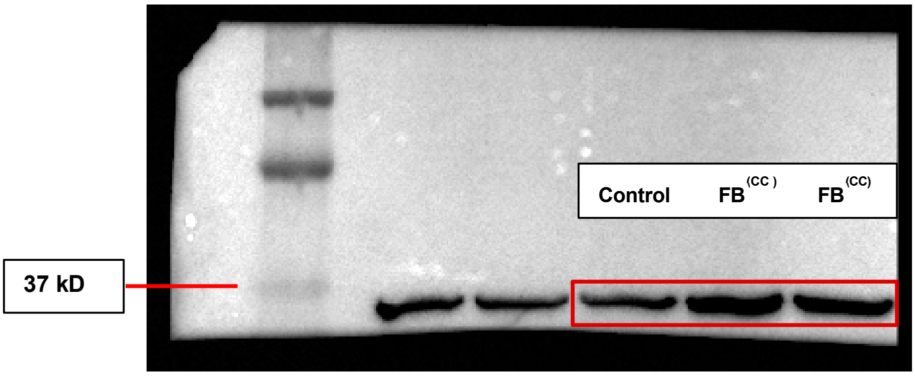

MCT1:

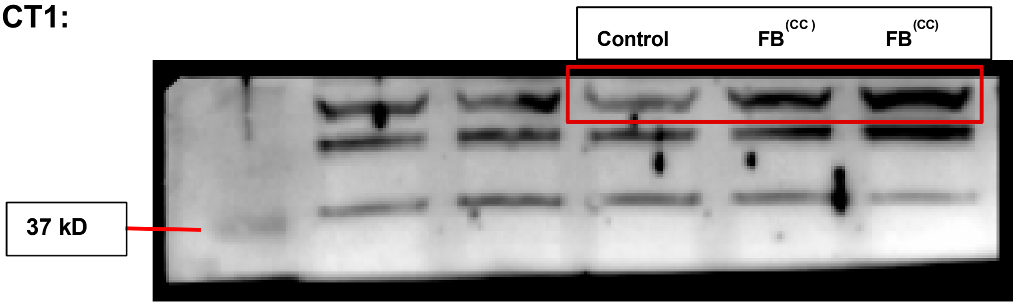

MCT4:

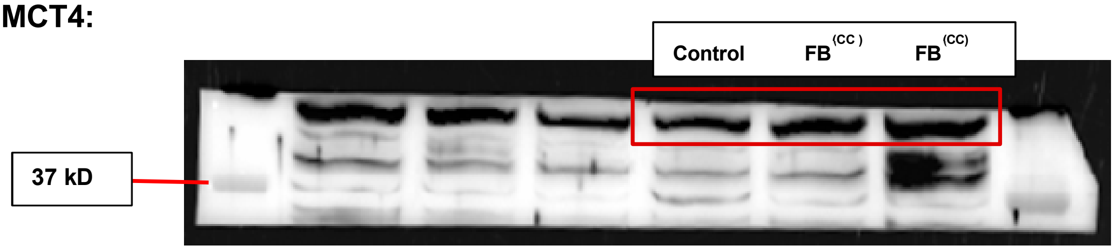

β-actin:

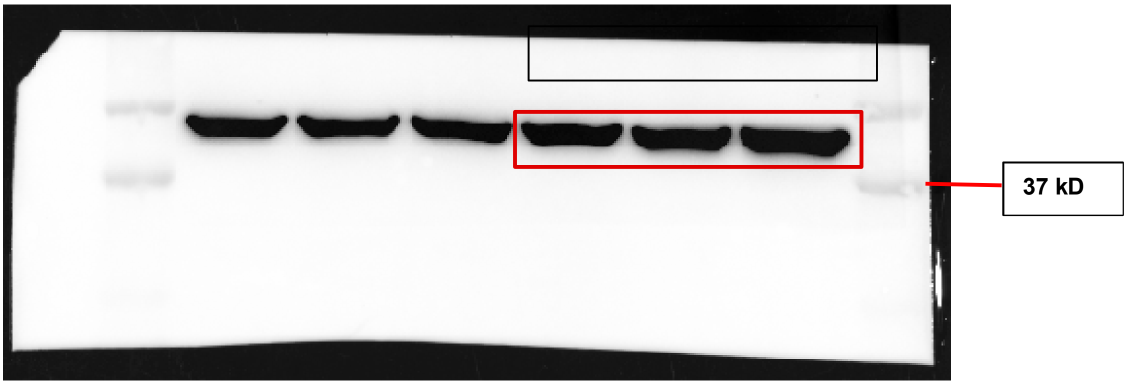

Supplement: Supplementary file 1 [file cancers-18-00294-s001.zip › cancers-4060287-supplementary.pdf]
